# Study on the Role of an Erythrocyte Membrane‐Coated Nanotheranostic System in Targeted Immune Regulation of Alzheimer's Disease

**DOI:** 10.1002/advs.202301361

**Published:** 2023-04-19

**Authors:** Yanrong Su, Yufen Huang, Qinjie Kou, Lu Lu, Haiye Jiang, Xisheng Li, Rong Gui, Rong Huang, Xueyuan Huang, Jinqi Ma, Jian Li, Xinmin Nie

**Affiliations:** ^1^ Department of Laboratory Medicine The Third Xiangya Hospital Central South University No.138,Tongzipo Road,Yuelu District Changsha Hunan 410013 China; ^2^ Department of Blood Transfusion The Third Xiangya Hospital Central South University No.138,Tongzipo Road,Yuelu District Changsha Hunan 410013 China; ^3^ Hunan Engineering Technology Research Center of Optoelectronic Health Detection Changsha Hunan 410000 China

**Keywords:** A*β* clearance, Alzheimer's disease, erythrocyte membrane, immune regulation, nano‐system

## Abstract

Alzheimer's disease (AD) is one of the most common neurodegenerative diseases in the elderly population. Despite significant advances in studies of the pathobiology on AD, there is still no effective treatment. Here, an erythrocyte membrane‐camouflaged nanodrug delivery system (TR‐ZRA) modified with transferrin receptor aptamers that can be targeted across the blood–brain barrier to ameliorate AD immune environment is established. Based on metal‐organic framework (Zn‐CA), TR‐ZRA is loaded with CD22shRNA plasmid to silence the abnormally high expression molecule CD22 in aging microglia. Most importantly, TR‐ZRA can enhance the ability of microglia to phagocytose A*β* and alleviate complement activation, which can promote neuronal activity and decrease inflammation level in the AD brain. Moreover, TR‐ZRA is also loaded with A*β* aptamers, which allow rapid and low‐cost monitoring of A*β* plaques in vitro. After treatment with TR‐ZRA, learning, and memory abilities are enhanced in AD mice. In conclusion, the biomimetic delivery nanosystem TR‐ZRA in this study provides a promising strategy and novel immune targets for AD therapy.

## Graphical abstract

An erythrocyte membrane coated bionic nanoparticle (TR‐ZRA) with targeted aptamer is designed. The nanoparticle can be targeted into the blood–brain barrier to regulate genes in microglia, enhance the ability to phagocytose A*β*, inhibit the level of complement activation, and monitor A*β* in vitro low‐costly and rapidly, providing a new strategy for Alzheimer's disease treatment.

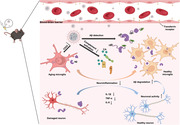

## 1 Introduction

Alzheimer's disease (AD) is gradually becoming the leading cause of dementia worldwide, with the prevalence of AD gradually increasing from 5.0% in people aged 65–74 years to 33.2% in people aged 85 years and older, resulting in more than 52 million people being affected worldwide.^[^ ^1^ ^]^ The pathogenesis of AD remains complex, but the most common explanation in recent studies is the amyloid cascade hypothesis.^[^ ^2^, ^3^ ^]^ The formation of A*β* plaques results from a series of amyloid peptides that are abnormally sheared. Excessive A*β* plaque accumulation could disrupt brain microvascular endothelial cells, impair the integrity of the blood‒brain barrier, and induce infiltration of innate immune cells and adaptive immune cells from outside the brain, resulting in disorders of the immune microenvironment in the brain.^[^ ^4^, ^5^ ^]^ Therefore, remodeling the immune microenvironment and eliminating the accumulation of abnormal A*β* in the brain is one of the most important strategies for the treatment of AD.

Microglia are macrophages in the brain and spinal cord, and they represent the primary line of immune defense in the central nervous system, as they are mainly responsible for clearing damaged neurons, A*β* plaques, and infectious substances in the central nervous system.^[^ ^6^ ^]^ However, aging microglia in the brains of AD patients may become dysfunctional and metabolically impaired, and their ability to clear A*β* is reduced, which results in the formation of a large number of A*β* plaques.^[^ ^7^, ^8^ ^]^ On the other hand, microglia in the AD brain show excessive activation and an uncontrolled state, and they can release many proinflammatory factors (such as tumor necrosis factor TNF‐*α*, interleukin IL‐1*β*, IL‐6, etc.) and complement components, further causing immune inflammatory damage.^[^ ^9^, ^10^ ^]^ At the same time, the membrane attack complex formed by complement activation can directly damage neurons that are highly sensitive to complement or stimulate microglia to release more inflammatory factors, forming a positive feedback loop to further aggravate immune inflammatory damage to neurons.^[^ ^11^ ^]^ In addition, proinflammatory mediators decrease the expression of receptors capable of binding to A*β* on the surface of microglia, which decreases A*β* phagocytosis by microglia and further promotes the production and deposition of A*β*.^[^ ^12^ ^]^ Therefore, modulating the immune response and enhancing microglial phagocytosis to promote clearance of A*β* while inhibiting complement to reduce immune inflammatory damage and synergistically protect neurons in the brain is a possible strategy to improve AD pathology.

In previous research, CD22 was found to be highly expressed in aging microglia.^[^ ^13^ ^]^ It was proposed that silencing CD22 might enhance A*β* clearance by microglia. Thus, an effective gene vector loaded with CD22 shRNA (RNAi) was designed to test this hypothesis. In addition, an A*β* aptamer (AAP), which can detect A*β* in the brain in real time, was designed to specifically bind to A*β* and indicate the content of A*β* via a fluorescence signal based on the fluorescence resonance energy transfer effect. However, the negative charge of the nucleic acid hinders its entry into the cellular cytoplasm. Moreover, these naked nucleic acid molecules are easily digested by intracellular nucleases.^[^ ^14^ ^]^ At present, the vectors for gene therapy mainly include viral vectors such as lentivirus, adenovirus, and adeno‐associated virus and nonviral vectors such as cationic liposomes, but due to their drawbacks of immunogenicity, oncogenicity, high costs, and a single function, they are difficult to translate to the clinic, and their applications are limited. Notably, metal‐organic frameworks (MOFs) are crystalline porous materials with periodic lattice structures that are formed via the self‐assembly of transition metal ions and organic ligands; these structures have high porosity and a large specific surface area and can adsorb plasmid DNA via noncovalent forces such as electrostatic interactions, hydrophobic interactions, and hydrogen bonding while protecting it from chemical and/or enzymatic degradation.^[^ ^15^, ^16^ ^]^ Chlorogenic acid (CA) is a phenolic acid extracted from honeysuckle that can be used as an organic ligand for the structure of metal‐organic framework (MOF） CA has antibacterial, antiviral, anti‐inflammatory, and complement activation inhibitory effects.^[^ ^17^ ^]^ Therefore, our research aimed to synthesize a Zn‐CA MOF by combining zinc ions (Zn^2+^) with CA for the efficient loading of CD22 shRNA and A*β* aptamer.

However, nanoparticles can be easily recognized and cleared as exogenous substances by the reticuloendothelial phagocytic system in the body.^[^ ^18^ ^]^ Red blood cells (RBCs), the most abundant blood cells without nuclei in mammals, are the main medium for oxygen delivery, and their cell membranes are easy to prepare.^[^ ^19^, ^20^ ^]^ Moreover, the self‐recognition molecule CD47 on the surface of erythrocytes endows the encapsulated nanoparticles with a long circulating half‐life and immune evasion function.^[^ ^21^, ^22^ ^]^ In addition, receptor‐mediated transcytosis is currently one of the most effective ways to mediate crossing of the blood‒brain barrier by biomolecules.^[^ ^23^ ^]^ Transferrin receptor (TfR), one of the most representative transcytosis receptors, was distributed widely in endothelial cells, plexus epithelial cells, neurons, and common glial cell in brain tissue. In this study, inspired by most reports, a TfR aptamer was designed as a TfR ligand.^[^ ^24^ ^]^ After binding to TfR, the nanosystem entered the blood–brain barrier through transcytosis of brain endothelial cells, and was then taken up by microglia, neurons, and other cells containing TfRs.^[^ ^25^, ^26^ ^]^Thus, the resulting erythrocyte‐camouflaged nanoparticles were expected to protect the whole system from degradation, and the TfR aptamer, which was attached to the surface of the erythrocyte membrane by lipid insertion, was predicted to improve the ability of the system to reach the blood‒brain barrier, both of which could play essential roles in enhancing efficacy.

**Figure 1:**
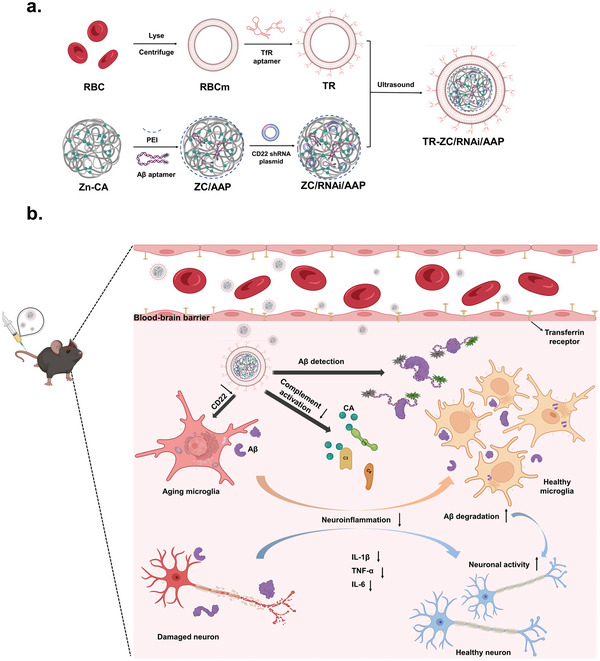
Schematic illustration of TR‐ZC/RNAi/AAPa）preparationand b) its regulation for AD therapy. After intravenous administration, TR‐ZRA can reach the blood–brain barrier and enter the brain environment, and then regulate the expression of CD22 in aging microglia, promote the phagocytosis and degradation of A*β* in microglia. CA inhibits complement activation and reduces neuroinflammation. AAP can monitor A*β* plaque in vitro. TR‐ZRA immunologically regulates Alzheimer's disease in different ways.

Hence, in this study, we proposed the use of Zn^2+^ and CA to construct a metal‐organic framework Zn‐CA (ZC), which simultaneously transports CD22 shRNA (RNAi) and A*β* aptamer (AAP) to form a ZC/RNAi/AAP (ZRA) nanocore. Finally, the TfR aptamer‐RBCm (TR) shell structure encapsulates ZRA to form a novel erythrocyte membrane‐camouflaged nanodrug system: TR‐ZC/RNAi/AAP(TR‐ZRA). As shown in **Figure** **1**a,b, TR‐ZRA was camouflaged by an autologous erythrocyte membrane and targeted to penetrate the blood‒brain barrier under the action of TfR aptamer. After phagocytosis and decomposition by microglia, ZC acts as an inhibitor of complement activation, and AAP indicates A*β* levels in the brain via fluorescence intensity. CD22 shRNA inhibits the expression of CD22 in microglia at the gene level and promotes the phagocytic clearance of A*β* by microglia, which further reduces immune inflammatory damage in the brain and improves neuronal activity. Thus, TR‐ZRA was designed to improve the immune environment of AD and provide a possible strategy to cure AD.

## 2 Results and Discussion

### 2.1 Preparation and Characterization of TR‐ ZC/RNAi/AAP

**Figure 2:**
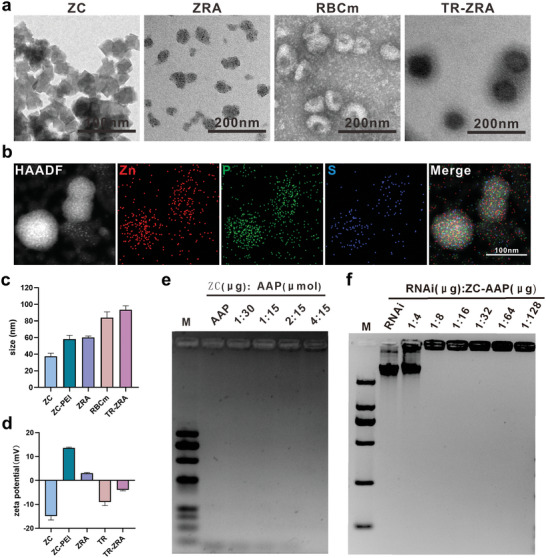
Characterization of the NPs. a) TEM image of ZC, ZRA, RBCm, and TR‐ZRA. Scale bar: 100 and 200 nm. b) The elemental mapping images of TR‐ZRA, scale bar: 100 nm. c) Size of NPs measured by TEM. d) Zeta potential of NPs detected by DLS. The appropriate ratio of e) ZC and AAP and f) ZC‐AAP and RNAi validated by gel image assay. Data are represented as the mean ± SD (*n* = 3).

The primary process of TR‐ZC/RNAi/AAP included incubation of Zn^2+^ and CA to synthesize a metal−organic framework (with PEI, positive charge) and loading with RNAi and AAP (negative charge), which resulted in the formation of ZC/RNAi/AAP nanocores via charge adsorption. The TfR‐A aptamer was inserted into erythrocyte membrane vesicles (RBCm) to form the T‐R shell structure. Finally, TR was used to encapsulate the ZC/RNAi/AAP nanocore and form TR‐ZC/RNAi/AAP by ultrasound (Figure 1a). Afterward, changes in the morphology, size, and zeta potential of these nanoparticles were measured. As shown in **Figure** **2**a, ZC exhibited 3D and quadrate crystal particles while ZRA was closer to the dispersed spherical structure, which may be caused by the modification of PEI, APP, and RNAi on the surface of ZC. TR‐ZRA was synthesized after ultrasonic treatment and filtration, and it can be seen that there was an erythrocyte membrane structure (≈8 nm) around ZRA. Moreover, the elemental mapping images of TR‐ZRA showed the co‐localization of Zn, the characteristic element of nanoparticles, in the elements (P and S) of RBCm, which also confirmed that ZRA was encapsulated by RBCm (Figure 2b). As shown in Figure 2c, the sizes of the ZC, ZRA, RBCm, and TR‐ZRA particles measured by TEM were ≈37.5, 60.1, 84.0, and 93.3 nm, respectively. In addition, as shown in Figure 2d, the zeta potentials of ZC and ZC‐PEI were −14.9 and 13.6 mV, respectively. After coincubation with the cationic polymerizer polyethyleneimine (PEI), ZC carried a positive charge on its surface, with a larger size and improved dispersion (Figure S1, Supporting Information). These results showed that the PEI modification of ZC caused a slight influence on the size, morphology, and potential of ZC, which were attributed to the characteristic of PEI.^[^ ^27^ ^]^ The dispersion of ZC was also improved because the positive charges repel each other, and this behavior can be employed to achieve binding of more nucleic acid molecules with negative charges to form ZRA.^[^ ^28^ ^]^ The TR, ZRA, and TR‐ZRA particles had values of −10.2, 3.05, and −4.0 mV, respectively(Figure 2d). It can be seen from the zeta potential changes of the particles that the plasmids and aptamers successfully combined with ZC, and TR was successfully attached to ZRA to synthesize TR‐ZRA. In addition, a gel electrophoresis assay was adopted to evaluate the complexation ability of CD22 shRNA and A*β* aptamer. As shown in Figure 2e, ZC‐AAP formation was limited to the initial gum hole when each micromole of AAP could combine with 0.27 µg or more ZC (the ratio was ≈4:15). In the same way, there was no free AAP or CD22 shRNA at the bottom of the gel when 1 µg CD22 shRNA was carried by 8 µg ZC‐AAP (with a surface‐mass ratio of 8:1) (Figure 2f). These results confirmed that ZRA could be synthesized step by step with the above proportions for subsequent experiments. Moreover, as shown in the UV−vis spectrum (Figure S2, Supporting Information), TR‐ZRA had absorption peaks at 280 and 405 nm, consistent with the characteristic absorption peaks of RNAi, AAP, and TR, respectively. SDS‒PAGE (Figure S3, Supporting Information) showed that TR‐ZRA possessed a protein profile similar to that of the erythrocyte membrane. DSPE‐PEG‐TfR‐A‐FAM was employed to prove that the targeting molecule TfR‐A could voluntarily insert into the surface of the erythrocyte membrane. As shown in Figure S4a, Supporting Information, green fluorescence was apparent on the surface of the erythrocyte membrane, which confirmed inlaying of the targeted probe TfR‐A onto the surface of the erythrocyte membrane via lipid insertion. And in Figure S4b, Supporting Information, it was observed that most of the erythrocyte membrane with green fluorescence was co‐located with RhB‐nanoparticles. Therefore, the obtained nanoparticles might exhibit the characteristics of immune escape and a long circulation period, which would increase the chances of passage through the BBB.^[^ ^29^ ^]^

### 2.2 Biocompatibility of the Nanoparticles

**Figure 3:**
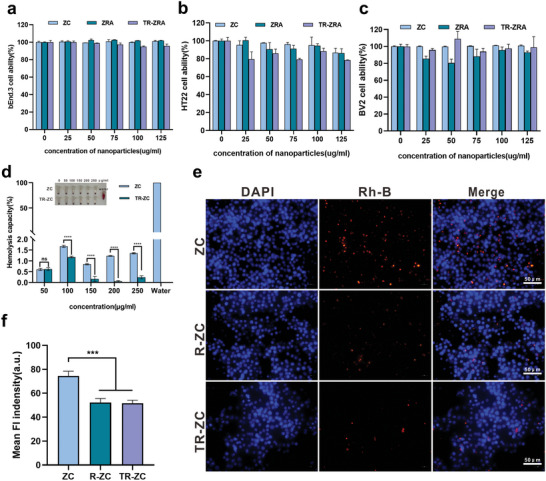
Biocompability of NPs. a–c) CCK8 assay to evaluate the cell viability of various concentrations of NPs toward bEnd.3, HT22 and BV2 cells for 24 h incubation. d) Hemolysis test results of NPs after incubation with 5% of the mice's RBC suspension for 2 h at 37 °C. The normal saline was used as a negative control and water was used as a positive control. ****p < 0.0001 determined by *t*‐tests. e) Representative fluorescence image of RAW 264.7 after incubation with different nanoparticles for 6 h at 37 °C, scale bar: 50 µm. f) Quantification of mean fluorescence intensity from (e). ***p < 0.001 determined by one‐way ANOVA and Tukey post hoc tests. Data are represented as mean ± SD (*n* = 3).

The biosafety of nanoparticles is vital for future applications. The cytotoxicity of the nanoparticles was evaluated by a standard Cell Counting Kit‐8 (CCK8) assay. The nanoparticles were incubated with various types of cells, including mouse brain endothelioma cells (bEnd.3), HT22 cells, and BV‐2 cells. After 24 h of incubation, all these cells retained >80% viability with different nanoparticles at concentrations from 0 to 125 µg mL^−1^ (**Figure** **3**a–c). In addition, we incubated RBCs from mice with different concentrations of ZC and TR‐ZC (0–250 µg mL^−1^) for 2 h at 37 °C to evaluate the safety of the nanoparticles in the blood circulation. As shown in Figure 3d, no significant hemolysis (less than 2%) was observed after incubation of ZC or TR‐ZC with RBC suspension. In addition, when ZC was coated with RBC membrane, the degree of hemolysis was further reduced, indicating that the ZC nanoparticles had good biological safety, and the erythrocyte membrane provided a further guarantee of the function of the nanoparticles in the blood circulation. Nanodrugs safely reach the target site and avoid being degraded by the immune system, which is one of the challenges for the effective delivery of nanomedicines to the central nervous system. We incubated mouse RAW 264.7 macrophages with ZC, ZC, and TR‐ZC. As shown in Figure 3e,f, we found that R‐ZC and TR‐ZC can evade phagocytosis by immune cells due to camouflaging by the erythrocyte membrane. It is possible that erythrocyte membrane proteins, such as CD47, mediated the long‐term circulation of the nanoparticles in the experiment by interacting with phagocytic receptors to escape phagocytic cells.^[^ ^30^, ^31^ ^]^ Therefore, more nanoparticles had the opportunity to cross the BBB. In our system, CD22 shRNA, aptamers, and RBCs were endogenous materials, and ZC was also safe, so TR‐ZRA had little effect on nerve cells, highlighting the advantages of this system as a therapeutic agent for AD therapy. Therefore, the biocompatibility of these nanoparticles in vitro confirmed their suitability for additional in vitro and in vivo experiments.

### 2.3 Establishment of Blood‒Brain Barrier and Penetration of Nanoparticles In Vitro

**Figure 4:**
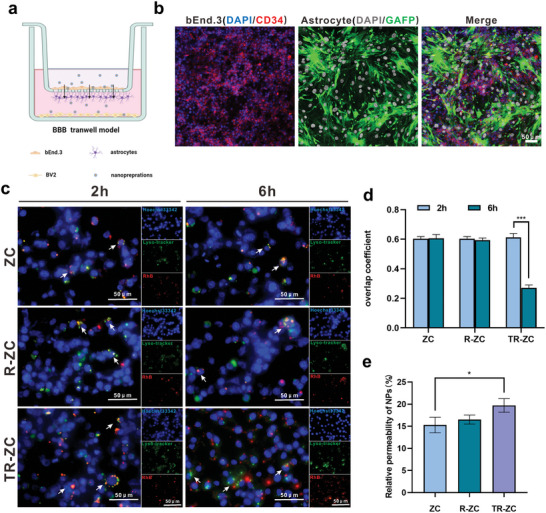
Establishment of blood‒brain barrier and penetration of nanoparticles in vitro. a) The schematic illustration of BBB transwell model in vitro. b) The CLSM fluorescence images after building the BBB model for 24 h, which contained bEnd.3 (nucleus: blue, CD34: red) and astrocyte (nucleus: gray, GFAP: green); scale bar: 50 µm. c) The endo/lysosomal escape of various NPs was assessed by CLSM after 2 and 6 h incubation, respectively; scale bar: 50 µm. *P < 0.05 determined by *t*‐test. d) Overlap coefficient of lyso‐tracker (green) and RhB‐NPs (red) of Figure 3c at 2 and 6 h was quantified by Image J software. e) The relative permeability of various NPs after incubation for 24 h in BBB model. ***P < 0.001 determined by one‐way ANOVA and Tukey post hoc tests. Data are presented as the mean ± SD (*n* = 3).

The blood‒brain barrier (BBB) contributes substantially to maintaining the stability of the internal environment around the brain tissue and preventing harmful substances from entering the brain tissue. The tight junctions established between endothelial cells limit the passage of many neurotherapeutic drugs across the BBB.^[^ ^32^ ^]^ In addition, the size, lipid solubility, and other physical and chemical properties of a drug also limit its ability to cross the BBB. Therefore, we next explored whether these synthetic nanoparticles could cross the blood‒brain barrier, which was simulated with a Transwell model in vitro. bEnd.3 and astrocyte cells were seeded in the upper and lower chambers, and BV2 was seeded in the bottom wells to simulate the BBB (**Figure** **4**a). After 12–24 h of inoculation, confocal laser scanning microscopy (CLSM) was used to verify the construction of the BBB model in vitro. As shown in Figure 4b, bEnd.3 and astrocyte cells were labeled with Cy 3 and FITC, respectively, and their confluence was 90% on both sides of the insert, which was considered to indicate successful BBB construction.

How to allow medicines with therapeutic effects within the central nervous system to transfer across the blood‒brain barrier (BBB) remains a challenge. Cerebral microvascular endothelial cells are regarded as some of the most important parts of the blood‒brain barrier, and they limit the flow of soluble substances and cells from the blood into the brain. The abundant expression of TfRs on most nerve cells made it possible for nanomaterials to cross the blood–brain barrier (Figure S5, Supporting Information). With the help of TfR aptamer, a large number of nanoparticles could be selectively transported into the brain environment via nanoparticle‐specific targeting mediated by the TfR on bEnd.3 cells. Therefore, RhB‐labeled nanoparticles were incubated with bEnd.3 cells for 2 and 6 h, and the localization of nanoparticles in the cells was observed by CLSM. As shown in Figure S6, Supporting Information, the cellular uptake of TR‐ZC was much greater than that of ZC and R‐ZC, which also indicated that TfR‐A could enhance targeted cellular uptake. Meanwhile, as shown in Figure 4c, after 2 h of incubation the red fluorescence of RhB‐nanoparticles mainly colocalized with the green fluorescence of endo/lysosomes as yellow spots in these groups. However, after incubation for 6 h, most of the red signals separated from the green signals in the TR‐ZC group, indicating that the nanoparticles had escaped from lysosomes. In contrast, the signals in the ZC and R‐ZC groups were still located in endosomes/lysosomes after 6 h of incubation. The overlap coefficient results were shown in Figure 4d. These results indicated that TfR aptamer‐TfR‐mediated trans endocytosis could be an effective drug‐specific delivery pathway that facilitates endo/lysosome escape and makes it possible for more nanoparticles to cross the blood‒brain barrier and be taken by microglial cells.

Therefore, after establishment of the BBB model, Cy5.5‐labeled nanoparticles were added to the upper culture medium to test their penetration. Specifically, the fluorescence intensity of equal quantities of different nanoparticles in the lower medium reflects their ability to cross the blood‒brain barrier in vitro (Figure S7, Supporting Information). As shown in Figure 4e, free ZC showed minimal intensity due to its lack of lipid solubility and poor BBB penetration. Upon coating with RBCm, however, the transport of ZC was slightly enhanced. Notably, the best BBB penetration efficacy was observed for TR‐ZRA due to the surface TfR aptamer modification that facilitated cell transportation via binding with TfR on bEnd.3 cells. The penetration in TR‐ZC was only slightly enhanced, which may be attributed to the short total observation time and the stationary superior culture medium in the in vitro blood–brain barrier model. Generally, these results suggested that TR‐ZRA can escape from bEnd.3 cells and efficiently cross the blood‒brain barrier, enabling further endocytosis by BV2 cells and regulation of microglial dysfunction.

### 2.4 Anticomplement Activity of Nanoparticles and A*β* Detection by A*β* Aptamer

**Figure 5:**
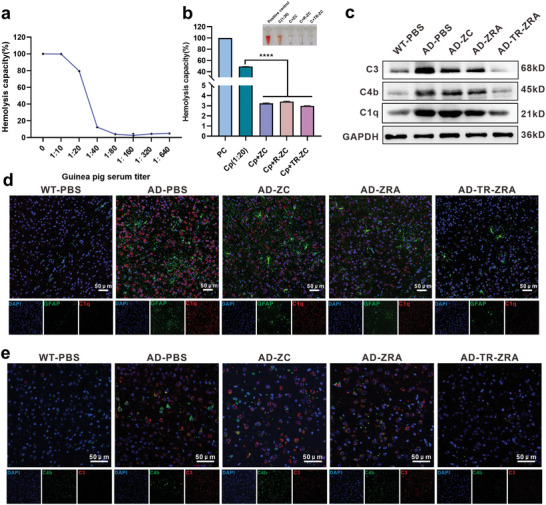
Anticomplement activity of NPs. a) The complement titer assessment of guinea pig serum by complement hemolytic assay in vitro. b) The hemolysis capacity of NPs after incubation with the guinea pig serum, the hemolysin, and the 2% SRBC at 37 °C for 30 min. PC: positive control; Cp: complement. c) Main complement C3, C4b, C1q expression in brain tissue evaluated by Western blot assay after different treatments in WT and AD mice. CLSM images of d) GFAP and C1q and e) C3 and C4b in brain sections after treatment of NPs in WT and AD mice; scale bar: 50 µm. ****p < 0.0001 determined by one‐way ANOVA and Tukey post hoc tests. Data are represented as the mean ± SD (*n* = 3).

The complement system is a double‐edged sword that is neuroprotective but can also be neurotoxic, depending on its starting target and activation level.^[^ ^33^ ^]^ It has been reported that the levels of C1q, C3, C4, and other complement components in the brains of rats with AD are higher, and these components largely colocalize with A*β* plaques in the brain, especially in the hippocampus and cortex, suggesting that these complement components may be related to neurodegeneration caused by neuroinflammation and synapse loss in the brain.^[^ ^11^ ^]^ As designed, ZC not only can be used as a drug transport carrier but also has anticomplement activity. The chlorogenic acid of ZC bound to proteins through hydrophobic interactions and hydrogen bonding and played a role in inhibiting complement activity.^[^ ^34^ ^]^ Fifty percent hemolytic inhibition through the classical pathway (CH 50) was used to evaluate the anticomplement activities. Guinea pig serum (complement) was diluted to 1:10, 1:20, 1:40, 1:80, 1:160, and 1:320 with PBS and added to the hemolysis system to evaluate titers. As shown in **Figure** **5**a, the hemolysis rate was close to 100% when the dilution of complement was 1:10–1:20, and the system achieved complete hemolysis. Guinea pig serum diluted 1:20 was selected to ensure complete hemolysis and improve the sensitivity of the system. As shown in Figure 5b, in the anticomplement assays, compared with the 1:20 diluted guinea pig serum group and the positive control, the ZC, R‐ZC, and TR‐ZC groups all displayed much lower hemolysis capacities, with values of 3.25%, 3.42%, and 2.99%, respectively. These results demonstrated that these treatments all possessed strong anticomplement activity, and their complement inhibition rates were up to 96.4%, 96.6%, and 96.8%, respectively. This result signified that the red cell membrane coating on the surface of the ZC did not affect the anticomplement activities of the ZC. Moreover, the anticomplement characteristics of ZC were verified in mice with AD. After nanodrug treatment for a month, Western blot (WB) assay showed that the content of complement components (C3, C4b, C1q) in the brain tissue of TR‐ZRA treated group was inhibited (Figure 5c and Figure S8, Supporting Information). Besides, as shown in Figure 5d,e and Figure S9a, Supporting Information, the red fluorescence representing the level of complement C1q around astrocytes (red fluorescence) was significantly reduced in the TR‐ZRA group but not in the ZC and ZRA groups. In addition, compared with that in the AD group, the fluorescence intensity of C3 and C4 was decreased in the TR‐ZRA group but the complement components of other experimental groups remained activated in the brain, which also reflected the inhibition of complement activity to a certain extent (Figure S9b, Supporting Information). Moreover, GFAP, the indicator of activated astrocytes, was also reduced, which indicated that the inflammation in brain was relieved to some extent (Figure S9c, Supporting Information). These results were slightly inconsistent with the in vitro results, which may be because TR‐ZRA, which reached the brain more abundantly under the action of targeting factors, fully reduced complement activity. In short, these experimental results indicated that the chlorogenic acid in the nanoparticles can inhibit the overactivation of complement in the brain environment and protect the nervous system.

**Figure 6:**
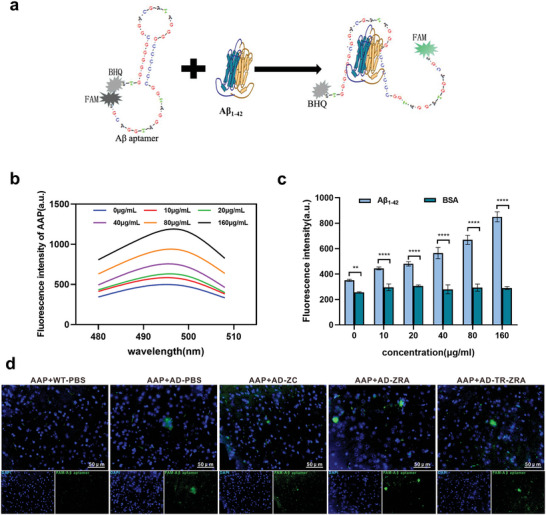
A*β* detection by A*β* aptamer. a) Schematic diagram of A*β* aptamer designed to detect A*β*_1‐42_. b) The fluorescence intensity change of AAP after incubation with different concentrations of A*β*_1‐42_ at 37 °C for 2 h. c) The fluorescence intensity of AAP after incubating with different concentrations of A*β*_1‐42_, with BSA respectively at 37 °C for 2 h, BSA was as negative control. d) Representative fluorescence images of the brain slice incubating with AAP at 37 °C for 2 h; scale bar = 50 µm. ** *p* < 0.01, and *** *p* < 0.001, ****p < 0.0001 determined by *t*‐test. Data are presented as mean ± SD (*n* = 3).

Nucleic acid aptamers, which are oligonucleotides that have high affinities and specificities for their targets because of their similarities as receptors, have received much attention.^[^ ^35^ ^]^ It is generally believed that these aptamers form secondary/tertiary structures that provide binding opportunities for their targets.^[^ ^36^ ^]^ In our design, the aptamer was folded into a secondary hairpin structure. In the absence of A*β*_1‐42_, fluorescence was quenched when the FAM group labeling the 3′ end was close to the BHQ group labeling the 5′ end. The aptamer could bind to A*β*_1‐42_ when A*β*_1‐42_ was present. FAM and BHQ then moved far away from each other, and the green fluorescence was restored (**Figure** **6**a). As shown in Figure 6b, after optimizing the experimental conditions, the relationship between the fluorescence intensity of AAP and the concentration of A*β*_1‐42_ was analyzed. The results showed that the fluorescence signal of AAP increased gradually with increasing A*β*_1‐42_ concentration, which indicated that AAP can indicate the A*β*_1‐42_ concentration in vitro under certain conditions (Figure S10a, Supporting Information). In addition, the same concentrations of A*β*_1‐42_ and BSA were incubated with different concentrations of AAP (0–160 µg mL^−1^) for 2 h at 37℃ to explore the reactivity of AAP (Figure 6c). Meanwhile, as shown in the Figure S10b, Supporting Information, different A*β* aggregates (A*β*_1‐42_, A*β*_1‐40_, A*β*_1‐20_) were respectively co‐incubated with aptamers at 37 °C for 2 h. The fluorescence intensity detected in A*β*_1‐42_ group was significantly higher than that in the other two groups, indicating that the designed fluorescence aptamers had strong specificity and could bind to the protein sequence of A*β*_1‐42_ for fluorescence recovery. As shown in Figure S10c, Supporting Information, the fluorescence intensity of AAP was both stable in the TBE and 10% FBS, which showed that the fluorescence intensity changed little within 24 h. AAP could also be used to monitor A*β* plaques in brain slices in vitro. We incubated 10 µm AAP with brain sections for 2 h at 37℃ and then observed the localization of A*β* on the brain sections under a fluorescence microscope. As shown in Figure 6d, AAP could specifically bind to A*β* plaques in brain slices from APP/PS1 mice and emit specific green fluorescence. Besides, the fluorescence image of AAP in the mice brain of TR‐ZRA group was observed directly, which showed a little obvious green signal compared with AD‐PBS group (Figure S10d, Supporting Information). Thus, these results indicated that this assay possesses the advantages of being economical and convenient and provides a new approach for monitoring A*β* in brain tissue in vitro.

### 2.5 CD22 Downregulation in Microglia and Degradation of Inflammatory Factors

**Figure 7:**
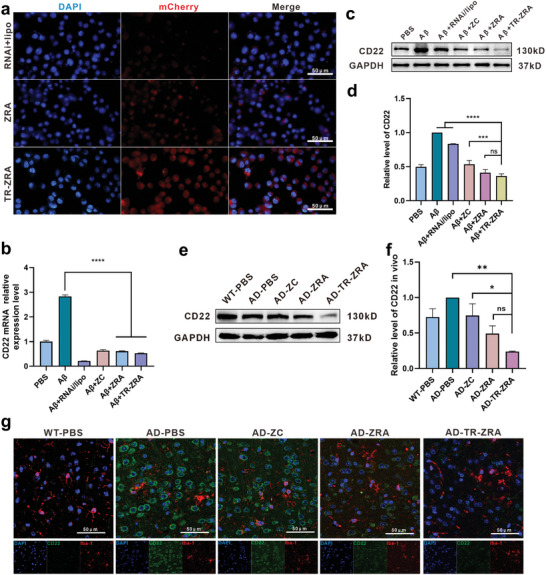
CD22 down‐regulation. a) Representative fluorescence images of BV2 cells after incubation with mCherry labeled RNAi+lipo, ZRA or TR‐ZRA at 37 °C for 24 h; scale bar: 50 µm. b) The CD22 mRNA levels in BV2 were quantified by qRT‐PCR after different treatments. PBS group was as control. c) CD22 expression in BV2 cell evaluated by Western blot assay after different treatments for 48 h at 37 °C and d) quantification results of CD22 expression in BV2. e) Western blot and f) quantified results of CD22 in the brain tissue of WT and AD mice with different treatments. AD‐PBS group was as control. g) Representative fluorescence images showing the expression of CD22 in brain tissue (IBAl: red and CD22: green; scale bar: 50 µm). * *p* < 0.05, ** *p* < 0.01, *** *p* < 0.001, ****p < 0.0001 determined by one‐way ANOVA and Tukey post hoc tests. Data are presented as mean ± SD (*n* = 3).

After simulating the BBB transport of the nanoparticles, we designed ^mCherry−^CD22shRNA to reduce CD22 expression in BV2 cell. We chose the commercial reagent Lipo8000 as the control. As can be seen from **Figure** **7**a and Figure S11, Supporting Information, the transfection efficiency of TR‐ZRA was more excellent in BV2 and the mean fluorescence intensity of the TR‐ZRA group was ≈1.56 times that of the ZRA group, which may be due to the action of targeting factors and erythrocytes membrane. There was no significant difference in plasmid delivery in normal and aging microglia (Figure S12, Supporting Information). Next, qRT‐PCR and Western blotting were employed to detect the expression of CD22 to determine the silencing efficacy of the nanoparticles. After treatment with A*β*_1‐42_, the expression of CD22 in BV2 cells was elevated. Compared with other groups, TR‐ZRA groups showed significant inhibition of CD22, indicating regulatory activity of delivered CD22shRNA. Among them, TR‐ZRA combined with targeting factors and erythrocyte membrane achieved higher delivery efficiency and thus had the strongest inhibitory effect on mRNA (**Figure** **7**b) and protein expression of CD22 in BV2 cells (Figure 7c,d). CD22 silencing by the nanoparticles was also demonstrated in mice. After 1 month of treatment, the brain tissue of mice was employed for WB and immunofluorescence assays. The WB results showed that the expression of CD22 in the AD‐TR‐ZRA group was significantly lower than that in the AD group, indicating that CD22 shRNA loaded with ZC effectively inhibited gene expression (Figure 7e,f). In addition, as observed in the immunofluorescence assay, CD22 expression (green) was significantly downregulated in IBA1‐labeled microglia (red) after TR‐ZRA treatment (Figure 7g). These results all demonstrated the higher silencing efficiency of TR‐ZRA, which can probably be attributed to TfR‐A's targetability and efficient transport of ZC. What's more, the activity of HT22 cells incubating with TR‐ZRA was investigated by Live/dead staining. As shown in Figure S13, Supporting Information, there was no significant difference in the fluorescence intensity of dead cells between the PBS and TR‐ZRA groups. These results indicate that CD22 silencing does not affect neuronal activity These results indicate that CD22 silencing could not affect neuronal activity.

**Figure 8:**
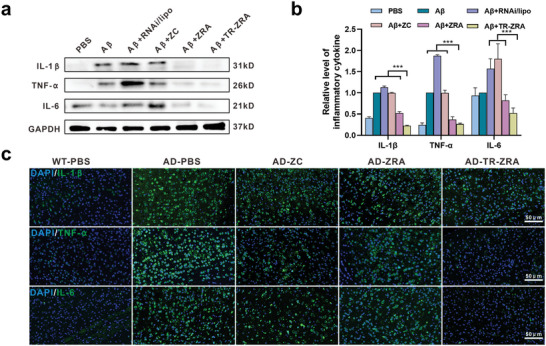
Degradation of inflammatory cytokine. a) IL‐1*β*, TNF‐*α*, and IL‐6 expression in BV2 evaluated by Western blot assay after different treatments for 48 h at 37 °C and b) quantification of inflammatory cytokine expression. c) Representative immunofluorescence images of inflammatory cytokines (IL‐1*β*, TNF‐*α*, IL‐6) expressed in WT and AD mice brain tissue; scale bar: 50 µm. *** *p* < 0.001 determined by one‐way ANOVA and Tukey post hoc tests. Data are presented as mean ± SD (*n* = 3).

Moreover, A*β*_1‐42_ can continuously stimulate microglial toxicity under the pathological condition of AD, resulting in the death of microglia and increased levels of proinflammatory cytokines, such as IL‐1*β*, IL‐6, and TNF‐*α*, in the brain. These proinflammatory factors in turn trigger and exacerbate A*β* aggregation, forming a vicious cycle during the pathological process and ultimately exacerbating AD.^[^ ^37^, ^38^ ^]^ Therefore, the levels of proinflammatory cytokines in BV2 cells were evaluated by Western blotting. As shown in **Figure** **8**a,b, we proved that the levels of IL‐1*β*, TNF‐*α*, and IL‐6 were obviously increased when BV2 cells were cocultured with A*β*_1‐42_ or the commercial transfection reagent Lipo, which might be associated with the toxicity caused by A*β*_1‐42_ or Lipo. The levels of these proinflammatory cytokines remained relatively high after ZC treatment alone, which might be due to the correlation between cellular uptake of the drug and the level of intracellular gene expression in aging BV2 cells. Nevertheless, the levels of these proinflammatory cytokines were decreased in the ZRA‐ and TR‐ZRA‐treated groups, which might be related to the CD22 level in BV2 cells.^[^ ^39^ ^]^ In particular, decreased CD22 levels could facilitate microglial status and promote phagocytosis of A*β*, which would further reduce inflammation levels. In addition, an immunofluorescence assay was adopted to detect typical proinflammatory factors, including IL‐1*β*, TNF‐*α*, and IL‐6 (green), in the cortex and hippocampus (Figure 8c and Figure S14, Supporting Information). The results showed that the levels of these cytokines were significantly reduced, confirming the anti‐inflammatory activity of the TR‐ZRA nanosystem. Neuroinflammation is mediated not only by the activation of glial cells (such as microglia) and the production of cytokines, chemokines, and ROS but also by the complement system. A*β* activates the NF‐*κ*B pathway, leading to increased release of C3, which can bind to C3aR on neurons and microglia, activate microglia, and induce the release of proinflammatory cytokines.^[^ ^40^ ^]^ C1q binding to A*β* and tau proteins in the AD brain can also cause complement activation, which leads to neuroinflammation and neurodegeneration in AD patients.^[^ ^41^, ^42^ ^]^ Thus, complement levels may decrease, and the associated neuroinflammation may also be relieved after TR‐ZRA treatment. Hence, the inflammatory environment in the AD brain was alleviated after TR‐ZRA treatment.

### 2.6 A*β* Degradation and Increased Neuronal Activity

**Figure 9:**
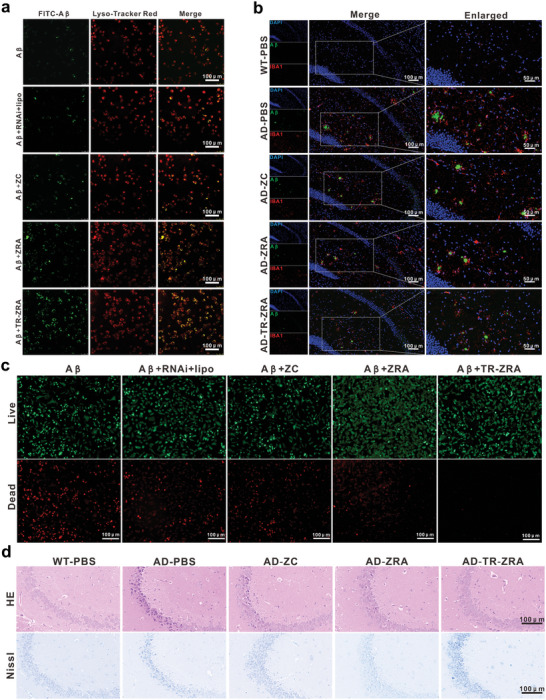
A*β* degradation and increased neuronal activity. a) Colocalization of FITC‐A*β*_1−42_ and lysosomes in BV2 after incubation with different preparations for 6 h. BV2 treated with FITC‐A*β*_1−42_ alone were used as a control. (Scale bar: 100 µm). b) Representative fluorescence images and enlarged vision images showing the location of microglia and A*β* plaque in brain tissue slice.(IBA1: red and A*β*: green); scale bar: 100 and 50 µm).c) Live/dead staining fluorescence images of HT22 cells after incubating with BV2 cells culture medium (after incubation with different preparations for 24 h) for 24 h; scale bar: 100 µm. d) Representative images of HE staining and Nissl staining of WT and AD mice after the different treatments; scale bar: 100 µm.

Microglia, a crucial line of immune defense in the central nervous system, play an important role in maintaining immune homeostasis in the brain. In the AD brain, activated microglia have an impaired ability to phagocytose A*β*, resulting in A*β* accumulation and further intensifying brain inflammation.^[^ ^43^, ^44^ ^]^ Therefore, A*β* degradation in BV‐2 cells is crucial to improve the AD environment. To simulate the AD environment, BV2 cells were incubated with A*β*_1‐42_ for 12 h to induce an inflammatory state. To investigate the effect of nanoparticles on the phagocytosis of A*β* by BV2 cells, FITC‐A*β*_1‐42_ was co‐incubated with proinflammatory microglial cells in the absence or presence of different preparations, including ZC, ZRA, and TR‐ZRA. After 6 h, colocalization of the FITC‐A*β*_1‐42_ oligomer with endo/lysosomes was observed by CLSM. Endo/lysosomes are the main organelles of A*β* degradation in microglia. As shown in **Figure** **9**a and Figure S15, Supporting Information, FITC‐A*β*_1‐42_ oligomer and endo/lysosome were mostly colocalized as yellow spots in the TR‐ZRA group, indicating that the phagocytosis of A*β*_1‐42_ by BV2 cells was enhanced in the presence of CD22 shRNA, while ZC alone did not significantly improve the phagocytosis effect, which was consistent with the hypothesis that the inhibition of CD22 in microglia promotes the elimination of A*β* by microglia. A consistent trend was observed in the brain tissue of AD mice. To observe the capture and degradation of A*β* by microglia in vivo, microglia and A*β* labeled with an anti‐IBA1 antibody and anti‐A*β* antibody, respectively, were analyzed by immunofluorescence. Compared with observations in WT mice, aggregation of the A*β* green fluorescence signal was obviously observed in AD mice suggesting microglial dysfunction of A*β* phagocytosis in AD mice (Figure 9b and Figure S16a, Supporting Information). In addition, in ZC‐ and ZRA‐treated AD mice, a mass of A*β* plaques and activated microglia were both observed, indicating that these nanoparticles could not reach the brain efficiently enough to encourage microglia to phagocytose A*β*. However, in the TR‐ZRA groups, only a few A*β* plaques were observed, and the area of activated microglia decreased in the brain (Figure S16b,c, Supporting Information). These phenomena suggested that after treatment with nanoparticles, the expression of CD22 in microglia was downregulated, which could enhance the dysfunction of healthy microglia, alleviate activated microglial infiltration and promote phagocytosis of A*β* in the brain tissue of AD mice. In conclusion, TR‐ZRA, compared with the other treatments, significantly ameliorated A*β* clearance and microglial dysfunction in the brain tissue.

It is generally considered that aggregated A*β*_1‐42_ can lead to cytotoxicity.^[^ ^45^ ^]^ It has been proven that TR‐ZRA can downregulate CD22 in microglia, which could improve the ability of BV2 cells to phagocytose A*β* and reduce the toxicity of A*β* to neurons. We performed live/dead staining to investigate the effect of the nanoparticles on A*β*‐induced cytotoxicity. BV2 cells were treated with A*β*_1‐42_ in the absence or presence of nanoparticles. After 24–48 h, the culture medium of BV2 cells was removed and incubated with HT22 cells for 24 h to evaluate cell activity. As shown in Figure 9c and Figure S17, Supporting Information, there were a number of dead cells after incubation with A*β*_1‐42_ alone for 24 h. In addition, free ZC or CD22 shRNA had little effect due to their poor cellular uptake and A*β* clearance. Nevertheless, the number of dead cells decreased significantly after treatment with TR‐ZRA medium, which means that the cells’ functions had been improved. This indicated that the TR‐ZRA nanoparticles could reduce A*β*‐induced cytotoxicity and promote cell proliferation. Similarly, both HE staining and Nissl staining showed improvements in neuron health after TR‐ZRA treatment. As shown in Figure 9d, the nucleoli became clearer, the cells were arranged neatly, and the intracellular Nissl bodies were more abundant, indicating that the neurons were repaired effectively.

### 2.7 Biodistribution, Magnetic Resonance Imaging, and Improved Memory Capability In Vivo

**Figure 10:**
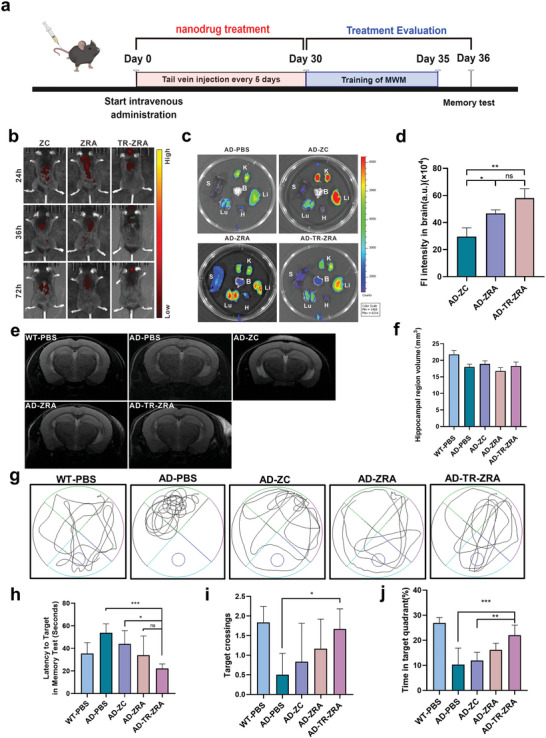
Biodistribution, magnetic resonance imaging, and improved memory capability in vivo. a) The schedule of treatment in mice. b) In vivo imaging of Cy5.5 labeled nanoparticles in APP/PS1 AD mice. c) Ex vivo imaging of main organs (heart, liver, spleen, lung, kidney, and brain) within 72 h after injection of different nanoparticles. d) The quantitative fluorescence intensity in the brain (*n* = 3). e) Representative MRI images and f) hippocampal region volume of every group of mice (*n* = 3). g) Representative swimming trajectories of mice in MWM. h) The time of latency to target platform on the test day in MWM, i) the times of crossing over the platform, and j) the percentage of time in the targeted quadrant of different groups (*n* = 6). *p < 0.05, **p < 0.01, *** *p* < 0.001 determined by one‐way ANOVA and Tukey post hoc tests. Data are presented as mean ± SD.

To evaluate the therapeutic effect of the nanoparticles in vivo, 6‐month‐old APP/PS1 mice were intravenously injected every 5 days for a month (**Figure** **10**a). To observe the distribution of nanoparticles in vivo, we injected Cy5.5‐labeled nanoparticles intravenously into mice. As expected, after intravenous administration, fluorescence images showed that TR‐ZRA group showed a strong fluorescence signal in the brain tissue in vivo 72 h after injection (Figure 10b). Besides, we observed the circulation of RhB labeled nanoparticles in mice. As shown in the Figure S18, Supporting Information, free ZC was rapidly cleared from the mouse after injection through the tail vein with a brief half‐life. In contrast, for R‐ZC, a significant increase in half‐life was observed. It should also be noted that the amount of nanoparticles in the TR‐ZC group also showed a rapid decrease in the amount of circulating in vivo with the shorter half‐life, but the amount in vivo was still relatively higher than that of free ZC. This result may be related to the loading of targeted factors on the nanoparticles after coating, which made most of the nanoparticles concentrate in the brain. This is mainly attributed to the TfR‐A aptamer and erythrocyte membrane modification on the nanoparticles, which helped TR‐ZRA target endothelial cells in the mouse brain and prolong the circulation in vivo, respectively. 72 h after injection, these mice were sacrificed by the cervical dislocation method. Their brain, heart, liver, spleen, lung, and kidney tissues were collected for fluorescence imaging in vitro, and the results were consistent with the previous results. As shown in Figure 10c, the ZC and ZRA groups showed a small amount of aggregation in the brain, liver, and kidney, while the accumulation of TR‐ZRA in the brain was significantly higher than that in the other groups. Compared to ZC and ZRA, TR‐ZRA showed 2.0/1.24‐fold‐enhanced Cy5.5 signals respectively 72 h after injection, indicating a stronger ability to target and penetrate the BBB (Figure 10d). These results indicated that TR‐ZRA had a great targeting effect to brain tissue in vivo.

Magnetic resonance imaging (MRI) can clearly display anatomical structures with high resolution, distinguish gray matter from white matter, and measure the area and volume of hippocampal structures in the brain, which means that MRI studies have been important for the clinical identification of AD. After a month of treatment, the brains of WT and AD mice were imaged by MRI. As shown in Figure 10e,f, a reduction in hippocampal region volume was observed in AD mice (17.985 mm^3^) relative to healthy WT mice (21.8 mm^3^), which was consistent with previous reports.^[^ ^46^, ^47^ ^]^ However, after TR‐ZRA treatment for a month, there was a slight upward trend in hippocampal volume (18.29 mm^3^), which might also be related to the amount of drug reaching the brain and the duration of treatment. This demonstrated that therapeutic components, such as chlorogenic acid and RNAi, have the potential to protect neurons, alleviate neuroinflammation and remove more A*β*.

On the other hand, the Morris water maze (MWM) experiment is an experiment in which mice are forced to swim and learn to find hidden platforms in the water. It is mainly used to test experimental animals' learning and memory ability in the context of sensing spatial position and direction. Therefore, the MWM experiment was employed to compare the spatial learning and memory abilities of the mice after a month of treatment. As shown in Figure 10g, the typical swimming track route and the number of target platform passes in each group of mice were recorded. On the test day, the platform was removed, and AD mice treated with PBS had severe memory deficits and displayed no significant improvement after 5 days of spatial learning (Figure S19, Supporting Information). However, AD mice treated with TR‐ZRA displayed substantially shorter escape latencies to target, with a 58.7% decrease compared with the PBS‐treated group (Figure 10h). In addition, compared with the other groups, the TR‐ZRA group crossed the platform more times (1.7 times) and spent more time (22.1%) in the target quadrant (Figure 10i,j). These behavioral experimental results indicate that TR‐ZRA could alleviate memory impairment in AD mice to a certain extent. Moreover, when all treatments were finished, the major organs of the mice were analyzed by HE staining (Figure S20, Supporting Information). The nanoparticles did not cause any pathologic changes in the major organs in these mice, indicating clear biocompatibility in vivo after 4 weeks of treatment via intravenous injection.

## 3 Conclusion

In conclusion, the BBB‐penetrating nanodrug system described here regulates the brain immune microenvironment in AD by enhancing microglial phagocytosis and inhibiting complement activation. It was found that TR‐ZRA is a nanoscale agent and has satisfactory biosafety for cells, the blood‒brain barrier, and various tissues and organs in in vivo and in vitro experiments. The chlorogenic acid carried by the nanosystem was confirmed to have the ability to modulate complement levels in the brain environment in AD, which was important for alleviating immune inflammatory damage. In addition, TR‐ZRA regulates CD22 levels in cells by delivering CD22 shRNA, which promotes microglial phagocytosis of A*β*, reduces the A*β* burden in cells and AD mice, inhibits microglial neuroinflammation, and facilitates memory capacity in AD mice. Thus, chlorogenic acid has synergistic effects with CD22 shRNA in regulating the immune microenvironment in the brain. In addition, the A*β* aptamer contained in TR‐ZRA was shown to be appropriate for in vitro monitoring of A*β*, which also provides a new way to observe A*β* plaques in the brain. These results suggested that TR‐ZRA has a broad modulatory effect, and thus, this nanodelivery system offers new possibilities for the treatment of brain aging and other neurodegenerative diseases, including but not limited to AD.

## 4 Experimental Section

### Materials

Chlorogenic acid (327‐97‐9) and ZnNO3 (10196‐18‐6) were purchased from Aladdin (Shanghai, China). DSPE‐PEG2000‐NH_2_ (R‐0038) was obtained from Xi'an Ruixi Biological Technology (China). Cell Counting Kit‐8 (CCK8) (K1018) was purchased from Apexbio (China). DAPI (C0060), Hoechst 33342 (C0031), RIPA lysis buffer (R0010), sheep erythrocyte hemolysin (H8360), 4% sheep red blood cell (SRBC) (S9045), guinea pig serum (complement) (S4990), and Albumin Bovine V(A8020) were purchased from Solarbio Life Science (China). The Calcein/PI Assay Kit (C2015S) and Lyso‐Tracker Red (C1046)/green (C1047S) fluorescent probe were purchased from Beyotime Biotechnology (China). A*β*_1‐42_ and FITC‐A*β*_1‐42_ were obtained from QYAOBIO (Shanghai, China). Dulbecco's modified Eagle's medium (DMEM), RPMI‐1640, and fetal bovine serum (FBS) were obtained from Procell (China). The cell culture chamber was purchased from Corning Incorporated (ME, USA). TfR aptamer (TfR‐A), A*β* aptamer (AAP), and CD22 shRNA plasmid (RNAi) were synthesized by Genechem Co., Ltd. Biotech (Shanghai, China).

Their sequences are as follows:

**Table:** 

TfR‐A aptamer	5″‐CGTAAATCAGTCAGAAGGCGTGGTACCACGCGCT/iFAMdT/TC‐3″
A*β* aptamer	5′ 6‐FAM1‐TGGGGGGCGGACGATAGGGGCCCCCCGGTAGGATGGAC G‐3′ BHQ1
CD22 shRNA‐a	5′‐CCCGGGCAACAAACTACACCTGGTATCTCGAGATACCAGGTGTAGT TTGTTGCTTTTTG‐3′
CD22 shRNA‐b	5′‐AATTCAAAAAGCAACAAACTACACCTGGTATCTCGAGATACCAGG TGTAGTTTGTTGC‐3′

### Cell Culture

bEnd.3 and HT22 cells were purchased from the Advanced Research Center, Central South University; BV2 cells were obtained from Procell (Wuhan, China). bEnd.3 cells were cultured in RPMI‐1640 medium supplemented with 10% fetal bovine serum. HT22 and BV2 cells were cultured in DMEM containing 10% fetal bovine serum (FBS) and 1% antibiotics (penicillin/streptomycin) at 37 °C in a 5% CO_2_ environment.

### Preparation of TR‐ZRA

First, fresh whole blood from mice was centrifuged at 3000 rpm for 10 min to gather the red blood cells. Cold PBS (0.25×) was added to lyse the erythrocytes and release hemoglobin. The cells were then repeatedly centrifuged five to six times at 5000 × *g* for 10 min to collect the erythrocyte membrane precipitate. Then, PBS (1.0×) was added for resuspension, followed by water bath sonication for 5 min. Finally, nanoscale erythrocyte membrane vesicles were extracted repeatedly with a syringe filter with a pore size of 0.45 µm.^[^ ^48^ ^]^ TfR‐A (10 µg), DSPE‐PEG2000‐NH 2 (10 mg), 1‐ethyl‐3‐(3′‐dimethyl aminopropyl)‐carbodiimide (EDC, 2.8 mg), and N‐hydroxysuccinimide (NHS, 1.7 mg) were dissolved in deionized water (5 mL) and stirred at 37 °C for 6 h. The carboxyl group in TfR‐A could be activated by EDC and NHS to form an amide bond with DSPE‐PEG2000‐NH_2_. Then, DSPE‐PEG2000‐NH_2_‐TfR‐A (0.1 mL) and erythrocyte membrane vesicles (0.9 mL) were blended at 37 °C for 6 h. TR was acquired by high‐speed centrifugation to eliminate dissociated molecules. Second, chlorogenic acid (0.8 g) and ZnNO_3_ (0.1 g) were dissolved in 5 mL of double‐distilled water. Chlorogenic acid was added dropwise into the ZnNO_3_ and stirred magnetically until the solution became milky white. The solution was then centrifuged, washed three times with double‐distilled water, and lyophilized into a powder with a vacuum freeze dryer for subsequent application. Third, ZC was resuspended in ultra‐pure water and treated with ultrasound for 10 min. The PEI polymer was then added to the nanoparticle solution and magnetically stirred overnight at room temperature to obtain ZC‐PEI nanoparticles, which were recovered by centrifugation (5000 × *g*, centrifugation at 25 °C for 20 min) and cleaned multiple times with ultra‐pure water to remove the unlinked PEI.^[^ ^49^ ^]^ CD22 shRNA and AAP were mixed with ZC‐PEI at different ratios, and the final volume of each solution was 20 µL. The solutions were incubated at room temperature for 30 min. Agarose gel electrophoresis was performed at 120 V for 20 min to observe the binding ability at different ratios of gene vector to plasmid. Finally, ZC/RNAi/AAP(ZRA) was constructed according to the optimal ratio. TR was ultrasonicated (42 kHz, 100 W) for 2 min with ZC/RNAi/AAP mixture to form TR‐ZC/RNAi/AAP.

### Characterization of Nanomaterials

The particle size distribution and zeta potential of the nanoparticles were measured using a Zeta‐sizer Nano ZS (Malvern Nano, Malvern, U.K.). The size and shape of the nanoparticles were measured by transmission electron microscopy (TEM). Membrane proteins of RBCm, TR, and TR‐ZRA were obtained with RIPA lysis buffer and quantified with a BCA protein assay kit (Beyotime, China). Sodium dodecyl sulfate‒polyacrylamide gel electrophoresis (SDS‒PAGE) was used to identify erythrocyte membrane proteins. Gels were stained with Komas Brilliant Blue staining reagent (Beyotime, China) and imaged with a gel imaging system (Bio‐Rad, USA).

### Cytotoxicity of the Nanoparticles

The cytotoxicity of the nanoparticles to cells was determined according to the manufacturer's instructions for CCK8. bEnd.3, HT22, and BV2 cells were added to 96‐well plates at a density of 1 × 10^4^ cells per well. After 24 h, 10 µL ZC, ZRA, and TR‐ZRA at different concentrations (0–250 µg mL^−1^) were added to each well, and the cells were incubated for 24 h. After 24 h, each well received 100 µL fresh medium and 10 µL CCK8 reagent for 2–4 h of incubation. Finally, the absorbance of each well was measured at 450 nm with a microplate reader. Cell ability (%) = [(As‐Ab)/(Ac‐Ab)] × 100%. As: Abs of the experimental hole; Ab: Abs of the blank hole; Ac: Abs of the control hole.

### Biocompatibility and Immune Escape Ability of the Nanoparticles

The hemocompatibility of nanoparticles was investigated by a hemolysis assay. Different concentrations (0–250 µg mL^−1^) of ZC and TR‐ZC were incubated with 5% erythrocytes of mice at 37 °C for 2 h. Then, the cells were centrifuged at 3500 rpm for 5 min, and the absorbance of the supernatant was measured at 545 nm using a microplate detector. Ultra‐pure water and PBS (pH 7.4) were used as positive and negative controls for hemolysis, respectively. The hemolysis rate was calculated from the measured absorbance. Hemolytic rate = 100% × [(OD_ex_‐OD_neg_)/(OD_po_‐OD_neg_)].OD_ex_: Abs of the experimental group; OD_neg_: Abs of the negative group; OD_po_: Abs of the positive group.

The immune escape ability of the nanoparticles was assessed with murine macrophages (RAW264.7). RAW264.7 cells (1 × 10^6^) were seeded in a 6‐well plate and cultured overnight. Then, ZC, R‐ZC, and TR‐ZC, with ZC 20 µg mL^−1^, were cocultured with the RAW264.7 cells for 6 h at 37 °C and 5% CO_2_. The free nanoparticles were discarded by washing them with PBS three times. The nuclei were stained with DAPI, followed by imaging via laser scanning microscopy.

### Primary Astrocyte Extraction

Primary astrocytes were isolated from the brains of neonatal 7‐day‐old mice according to previously described procedures.^[^ ^50^ ^]^ 7‐day‐old C57BL/6 female mice were sacrificed under aseptic conditions, and the brain tissue was removed from the ultraclean table into Hanks solution. The meninges and blood vessels were removed with pointed forceps, and the intact cerebral cortex was minced, added to trypsin, and then placed in a cell culture incubator (37 °C, 5% CO_2_) for 10 min after being mixed thoroughly. The samples were removed and mixed every 5 min until no obvious brain tissue clumps were observed. After the addition of DMEM‐F12 medium containing serum to terminate the digestion, the samples were filtered through a 0.44 µm sieve and centrifuged at 1000 rpm for 5 min. The supernatant was discarded, and the pellet was resuspended in fresh complete medium, added to culture dishes at a density of 1 × 10^7^ cells per well, and incubated at 37 °C and 5% CO_2_. The medium was changed every 2 days, and 7–10 days later, astrocytes with protruding shapes could be observed under the microscope. The extracted astrocytes were frozen for subsequent experiments.

### Construction of an In Vitro Blood‒Brain Barrier Model and Penetration of Nanoparticles

An in vitro BBB model was generated with a microporous Transwell membrane (0.4 µm) and cultured cells to investigate the ability of nanoparticles to penetrate the BBB, as described previously, with some modifications.^[^ ^51^ ^]^ Specifically, the upper side of the membrane was inoculated with bEnd.3 cells, and the lower side was inoculated with astrocytes. In addition, BV2 were inoculated into 6‐well plates to more accurately simulate the microenvironment in the brain. When the cell density reached 90% under the microscope, the model was considered to be established successfully, and A*β*_1‐42_ was added for further incubation for 48 h to simulate the pathological conditions of AD. ZC, Cy5.5‐NH_2_ or Rhodamine B‐PEG‐NH_2_, EDC, and NHS were dissolved in deionized water (5 mL) and stirred at room temperature for 6 h. The mixed system was centrifuged (at 5000 × *g*, 10 min) and rinsed four to five times with ultrapure water to remove free fluorescence. Centrifugation was reserved for precipitation, that is, fluorescently labeled nanoparticles, which were stored at 4 °C away from light. When the model was established successfully, Cy5.5‐ZC, Cy5.5‐ZRA, and Cy5.5‐TR‐ZRA were added to the upper chambers of the Transwell for coincubation with bEnd.3 cells, and the ZC concentration was 40 µg mL^−1^. After the addition of nanoparticles, 100 µL medium was collected every 6 h from the upper and lower sides of the filter membrane, the fluorescence intensity was detected with a multifunctional microplate reader, and the changes in fluorescence intensity were recorded. The penetration ability was evaluated by the ratio of fluorescence intensity in upper and lower culture medium at 24 h.

After the experiment, the filter membrane was cut off with scissors, and the cells on both sides of the membrane were washed three times with PBS for 5 min each time and then fixed with 4% paraformaldehyde at room temperature for 15–30 min. Then, 0.5% Triton‐100 was added for permeabilization at room temperature for 20 min, and BSA was added to terminate permeabilization at room temperature. Then, bEnd.3 cells and astrocytes were incubated with rabbit‐derived CD34 and GAFP primary antibodies (1:500) overnight at 4 °C and washed with PBS three times. Next, Cy3‐labeled goat anti‐rabbit secondary antibody (1:500) and FITC‐labeled goat anti‐rabbit secondary antibody (1:500) were incubated with bEnd.3 cells and astrocytes, respectively, for 1 h at 37 °C in the dark, followed by washing with PBS five times. Finally, the nuclei were stained with DAPI (1:5000), and the cells were incubated for ≈10–15 min at room temperature, washed with PBS, and then observed. Images were captured with a laser confocal fluorescence microscope.

### Cellular Internalization of bEnd.3 and BV‐2 Cells

A total of 1 × 10^6^ bEnd.3 cells were seeded in a 6‐well plate and cultured overnight at 37 °C. Then, Rh B‐ZC, Rh B‐R‐ZC, and Rh B‐TR‐ZC (with ZC 40 µg mL^−1^) were co‐incubated with bEnd.3 cells for 4 h. Next, the cells were washed with PBS three times to remove free nanoparticles and then fixed with 4% paraformaldehyde for 15 min, followed by staining with DAPI. To further probe whether TfR‐A mediates transcytosis of bEnd.3 cells, which could cause lysosomal escape of nanoparticles, Rh B‐ZC, Rh B‐R‐ZC, and Rh B‐TR‐ZC were incubated with bEnd.3 cells for 2 and 6 h. Then, the cells were washed with PBS (pH 7.4) three times and stained with Lyso‐Tracker green and Hoechst 33342. After washing with PBS three times, the cells were observed by CLSM.

To explore whether the nanoparticles can carry plasmids into cells, 1 × 10^6^ BV2 cells were seeded in a 6‐well plate and cultured overnight. Then, CD22 shRNA (RNAi)+lipo, ZC, ZRA, and TR‐ZRA (at an RNAi concentration of 2 µg) were co‐incubated with BV2 cells for 24 h. After two washes with PBS, the BV‐2 cells were fixed with 4% paraformaldehyde. The nuclei were stained with DAPI, and the cells were incubated for 10–15 min at room temperature and observed via CLSM.

### Quantitative Real‐Time PCR (qRT‒PCR)

Total RNA was extracted from BV2 cells using the Trizol method. cDNA was generated by reverse transcription. qRT‒PCR was performed using the Trans SYBR Green PCR Master Mix kit. GAPDH was used as an internal reference. The primer sequences were as follows:

CD22‐F: 5ʹ‐GATTCCATGCCTGTCAGCTG‐3ʹ;
CD22‐R: 3ʹ‐TGCACGAGACTCCCATTCTT‐5ʹ;
GAPDH‐F: 5ʹ‐CTAGGCCACAGAATTGAAAGATCT‐3ʹ; and
GAPDH‐R: 3ʹ‐ GTAGGTGGAAATTCTAGCATCATCC‐5ʹ.

### WB Assay

BV‐2 cells were added to 6‐well plates at a density of 8 × 10^5^ cells per well and incubated for 24 h. The cells were then co‐incubated with RNAi + Lipo, ZC, ZRA, and TR‐ZRA (RNAi 4 µg mL ^−1^) together with A*β*_1‐42_ oligomer in serum‐free DMEM for 48 h. Cells co‐incubated with A*β*_1‐42_ oligomer were used as controls. After incubation, the BV2 cells in each well were lysed with RIPA buffer, and the lysate was incubated on ice for 30 min. Then, the supernatant was collected by centrifugation for ≈10–15 min at 14 000 rpm and 4 °C. A BCA kit was used to measure the protein concentration. The proteins were separated by 10% SDS‐polyacrylamide gel electrophoresis, with a protein loading volume of 30 µg, and transferred to polyvinylidene fluoride membranes for blotting after electrophoresis. The blots were blocked with 5% skim milk in TBST at room temperature for 1 h. Different primary antibodies were added to the membranes separately and incubated overnight at 4 °C, followed by incubation with the appropriate secondary antibodies at room temperature for 1 h. The blots were developed using ECL technology. GAPDH was used as an internal reference for total proteins. Similarly, the expression levels of CD22, IL‐1*β*, IL‐6, and TNF‐*α* in the brain tissue of the WT and AD mice were detected by Western blotting. The animals were sacrificed by cervical dislocation, and half of the mouse brain tissue was ground and incubated with RIPA lysis solution. The lysate was collected and placed in a centrifuge at 4 °C and 14 000 rpm for ≈10–15 min, and the supernatant was collected. The rest of the procedure was the same as described above.

### Endocytosis of A*β* in BV‐2 Cells

BV2 cells (7 × 10^4^ cells per well) were added to a 35 mm laser confocal dish and incubated for 12 h. After the addition of fresh serum‐free medium, the cells were coincubated with FITC‐A*β*_1‐42_ (2 µg mL^−1^) together with different nanopreparations, including ZC, ZRA, and TR‐ZRA. After 6 h of incubation, the cells cultured in medium alone served as a negative control group. After incubation, the BV2 cells were washed with PBS three times and then stained with Lyso‐Tracker Red and Hoechst 33342. After staining, the cells were washed three times with PBS, and the uptake of FITC‐A*β*_1‐42_ by the cells was observed by CLSM at 492 nm (FAM), 577 nm (Lysotracker‐Red), and 346 nm (Hoechst 33342).

### Live/Dead Staining of HT22 Cells

As described above, BV2 cells were coincubated with A*β*_1‐42_ oligomer (10 µm) to induce a proinflammatory state in the BV2 cells. Then, the cells were coincubated with FBS‐free culture medium or different preparations for 36 h. Thereafter, the culture medium was collected and centrifuged (3000 rpm, 20 min) at 4 °C to collect the supernatant. Then, the supernatants from different wells were added to HT22 cells in 6‐well plates for 24 h, and the cells were stained with calcein‐AM and PI. Finally, the HT22 cells were observed via CLSM.

### Anticomplement Assessment In Vitro

Anticomplement experiments were performed using previously reported procedures.^[^ ^52^ ^]^ First, guinea pig serum was subjected to serial dilution (1:10, 1:20, 1:40, 1:80, 1:160, and 1:320) to determine the titer of complement. Then, the diluted complement was mixed with sheep erythrocyte hemolysin and 2% SRBC at 37 °C for 30 min. A group treated with 2% SRBC and water was used as a positive control, and PBS was used as a negative control. After determining the titer of complement, guinea pig serum, sheep erythrocyte hemolysin, 2% SRBC, and different preparations (ZC, R‐ZC, TR‐ZC) were added together and incubated at 37 °C for 30 min. These solutions without nanoparticle preparations were used as a positive control.

### Fluorescence Assay of A*β* In Vitro

A*β* aptamers were heated at 95 °C for 10 min and then gradually cooled to room temperature for 1 h in TBS buffer (1 mm Tris‐HCl, 100 mm NaCl, 4 mm MgCl_2_ and 5 mm KCl, pH 7.4) to form stable folded structures. Then, 2 µL A*β* aptamer solution (10 µm) and different concentrations of A*β* solution (0–160 µg mL^−1^) were mixed in TBS buffer in 96‐well plates. After incubation at 37 °C for 2 h, the fluorescence intensity was measured with a multifunction microplate analyzer, whose excitation wavelength and emission wavelength were set to 428 and 528 nm, respectively.

### Animals

APP/PS1 transgenic mice (female, 6–8 months old) and C57BL/6 wild‐type mice (female) were purchased from Beijing HFK Bioscience Co., Ltd. The animals were maintained in a pathogen‐free animal facility, with free access to food and water. This study was carried out in accordance with the relevant guidelines and regulations for the care and use of laboratory animals. The animal procedures were approved by the Animal Experimentation Ethics Committee of Central South University. (Approval number: 2020sydw0173).

### Treatment of Mice

The APP/PS1 mice were randomly divided into 4 groups (*n* = 3), and PBS, ZC, ZRA, and TR‐ZRA were injected into the tail vein every 5 days for a total of six times; the dose of RNA was 0.8 mg kg^−1^. Wild‐type C57BL/6 mice (WT) were treated with 200 µL saline via the tail vein every 4 days for a total of seven times as the control group.

### Morris Water Maze Experiment

The Morris water maze was used to investigate the spatial learning and memory abilities of mice. Specifically, the MWM system was a circular pool with a diameter of 120 cm, a height of 50 cm, a water temperature of ≈21–26 °C, and a water depth of 20 cm, which was divided into four quadrants as four entry points. The second quadrant was the target quadrant, and a circular platform with a diameter of 10 cm was placed in the target quadrant. A camera above the pool recorded the swimming tracks of the mice, and Smart 3.0 software was used to analyze the movement tracks of the mice. During the experiment, a few obvious markers were set on the pool wall to facilitate the memory of the mice.

Each mouse was trained four times a day and placed in the pool in different quadrants during the training period. The maximum time to find the platform was set to 60 s, and the time to find the platform was recorded as the incubation period. If a mouse could not find the platform, the incubation period was recorded as 60 s, and the mouse was guided to reach the platform and stay on the platform for 10–15 s. The training period lasted for 5 days, and the exploratory period began on the sixth day. The water platform was removed to test the spatial memory ability of the mice. Mice were placed in the opposite quadrant of the target quadrant, and the experiment lasted for 60 s. The latency to the platform, the number of times passing the platform, and the percentage of time spent were all recorded.

### Biodistribution Study

AD mice were injected intravenously with ^Cy5.5−^ZC, ^Cy5.5−^ZRA, and ^Cy5.5−^TR‐ZRA (1 mg kg^−1^ Cy5.5) and anesthetized. In vivo imaging experiments were performed at 24, 36, and 72 h after injection using an in vivo optical imager. To visualize the biodistribution of ^Cy5.5−^ZC, ^Cy5.5−^ZRA, and ^Cy5.5−^TR‐ZRA in vitro, mice were sacrificed 12 h after injection. Different organs were isolated and washed with PBS, and fluorescence images were captured in vitro. IVIS software was used for analysis.

### Magnetic Resonance Imaging

MRI experiments were performed on a 7.0T vertical bore Bruker Biospec 70/30 scanner (BrukerBioSpin MRI GmbH, Rheinstetten, Germany). The parameters used in the scans were optimized for gray‒white matter content. Based on reference multislice RARE scans (axial, sagittal, and coronal), with TR = 2500 ms, echo train length = 8, TEeff (echo time) = 36 ms, field‐of‐view (FOV, rectangle) = 20 × 20 mm and matrix size = 384 × 384 ×1 5, the voxel size was 0.052833209  × 0.052833209 × 0.5 mm. The total imaging time was 30 min.

### HE Staining and Nissl Staining

The experimental mice were sacrificed by cervical dislocation after 1 month of treatment, and the main organs of the animals, namely, the heart, brain, liver, spleen, lungs, and kidneys, were collected for HE staining. The major organs were fixed, dehydrated, paraffin‐embedded, serially sectioned, and stained with hematoxylin and eosin for light microscopic evaluation. Cresyl violet stain was used to observe neuronal damage via Nissl staining. The pathological changes in major organs were evaluated by three experienced pathologists according to “A Practical Guide to the Histology of the Mouse” written by Cheryl L. Scudamore (print ISBN: 9781119941200, online ISBN: 9781118789568, https://doi.org/10.1002/9781118789568).

### Immunofluorescence Assay

The immunofluorescence technique was performed according to standard protocols. Brain tissue sections were first washed with PBS and then blocked with normal goat serum for 1 h. Then, the brain tissue sections were incubated with the appropriate primary antibody overnight at 4 °C. The two kinds of primary antibodies were mixed at a specific dilution ratio and added to the tissue in drops. The sections were placed flat in a wet box and incubated at 4 °C overnight. After incubation with the primary antibodies, the slides were placed in PBS (pH 7.4) and washed three times for 5 min each. Then, labeled secondary antibodies from the corresponding species were added at a specific dilution and incubated with the tissue sections at room temperature for 50 min. The cells were washed with PBS (pH 7.4) three times for 5 min each. Then, the nuclei were stained with DAPI, and the samples were incubated for 10 min at room temperature in the dark. The slides were placed in PBS (pH 7.4) and washed three times with shaking on a decolorization shaker. The slices were slightly dried and sealed with an anti‐fluorescence quenching tablet. Finally, the sections were placed under a microscope to capture images (DAPI: Ex = 330–380 nm, Em = 420 nm; FITC: Ex = 465–495 nm, Em = 515–555 nm; CY3: Ex = 510–560 nm; Em = 590 nm).

### Statistical Analysis

All data were expressed as mean ± SD. The control group was taken as the benchmark, and the ratio between the experimental group and the control group was used to reflect the relative expression level between the groups. When the measurement was significantly above the normal value or when the deviation from the mean value in the measurement was more than two standard deviations, it was considered an outlier. For the processing of abnormal values, the data will be eliminated, or the median, average value, and so on will be used to fill in. For multiple‐group comparisons, one‐way ANOVA was employed followed by Turkey's test. Specific comparisons between the two groups were carried out with an unpaired Student's *t*‐test (two‐tailed). Statistical significance was shown as *p < 0.05, **p < 0.01, ***p < 0.001, and ****p < 0.0001. GraphPad Prism version 8.0 software was used for statistical analysis.

## Conflict of Interest

The authors declare no conflict of interest.

## Supporting information

Supporting Information

## Data Availability

Research data are not shared.

## References

[1] Alzheimer's Dementia 2022, 18, 700.

[2] J. A. Hardy , G. A. Higgins , Science 1992, 256, 184. 1566067 10.1126/science.1566067

[3] J. Hardy , D. Allsop , Trends Pharmacol. Sci. 1991, 12, 383. 1763432 10.1016/0165-6147(91)90609-v

[4] M. T. Heneka , M. J. Carson , J. El Khoury , G. E. Landreth , F. Brosseron , D. L. Feinstein , A. H. Jacobs , T. Wyss‐Coray , J. Vitorica , R. M. Ransohoff , K. Herrup , S. A. Frautschy , B. Finsen , G. C. Brown , A. Verkhratsky , K. Yamanaka , J. Koistinaho , E. Latz , A. Halle , G. C. Petzold , T. Town , D. Morgan , M. L. Shinohara , V. H. Perry , C. Holmes , N. G. Bazan , D. J. Brooks , S. Hunot , B. Joseph , N. Deigendesch , et al., Lancet Neurol. 2015, 14, 388. 25792098 10.1016/S1474-4422(15)70016-5 PMC5909703

[5] S. Jevtic , A. S. Sengar , M. W. Salter , J. McLaurin , Ageing Res. Rev. 2017, 40, 84. 28941639 10.1016/j.arr.2017.08.005

[6] H. Sarlus , M. T. Heneka , J. Clin. Invest. 2017, 127, 3240. 28862638 10.1172/JCI90606 PMC5669553

[7] S. Anwar , S. Rivest , Expert Opin. Ther. Targets 2020, 24, 331. 32129117 10.1080/14728222.2020.1738391

[8] G. J. Harry , Pharmacol. Ther. 2013, 139, 313. 23644076 10.1016/j.pharmthera.2013.04.013 PMC3737416

[9] S. Hong , V. F. Beja‐Glasser , B. M. Nfonoyim , A. Frouin , S. Li , S. Ramakrishnan , K. M. Merry , Q. Shi , A. Rosenthal , B. A. Barres , C. A. Lemere , D. J. Selkoe , B. Stevens , Science 2016, 352, 712. 27033548 10.1126/science.aad8373 PMC5094372

[10] D. Kaur , V. Sharma , R. Deshmukh , Inflammopharmacology 2019, 27, 663. 30874945 10.1007/s10787-019-00580-x

[11] A. Shah , U. Kishore , A. Shastri , Int. J. Mol. Sci. 2021, 22, 13647. 34948444 10.3390/ijms222413647 PMC8705098

[12] S. E. Hickman , E. K. Allison , J. El Khoury , J. Neurosci. 2008, 28, 8354. 18701698 10.1523/JNEUROSCI.0616-08.2008 PMC2597474

[13] J. V. Pluvinage , M. S. Haney , B. A. H. Smith , J. Sun , T. Iram , L. Bonanno , L. Li , D. P. Lee , D. W. Morgens , A. C. Yang , S. R. Shuken , D. Gate , M. Scott , P. Khatri , J. Luo , C. R. Bertozzi , M. C. Bassik , T. Wyss‐Coray , Nature 2019, 568, 187. 30944478 10.1038/s41586-019-1088-4 PMC6574119

[14] R. Acharya , S. Saha , S. Ray , S. Hazra , M. K. Mitra , J. Chakraborty , Mater. Sci. Eng., C 2017, 76, 1378. 10.1016/j.msec.2017.03.009 28482505

[15] J. Zhuang , C. H. Kuo , L. Y. Chou , D. Y. Liu , E. Weerapana , C. K. Tsung , ACS Nano 2014, 8, 2812. 24506773 10.1021/nn406590q

[16] W. Liang , P. Wied , F. Carraro , C. J. Sumby , B. Nidetzky , C. K. Tsung , P. Falcaro , C. J. Doonan , Chem. Rev. 2021, 121, 1077. 33439632 10.1021/acs.chemrev.0c01029

[17] S. F. Nabavi , S. Tejada , W. N. Setzer , O. Gortzi , A. Sureda , N. Braidy , M. Daglia , A. Manayi , S. M. Nabavi , Curr. Neuropharmacol. 2017, 15, 471. 27012954 10.2174/1570159X14666160325120625 PMC5543670

[18] N. Liu , M. Tang , J. Ding , Chemosphere 2020, 245, 125624. 31864050 10.1016/j.chemosphere.2019.125624

[19] C. M. Hu , L. Zhang , S. Aryal , C. Cheung , R. H. Fang , L. Zhang , Proc. Natl. Acad. Sci. U. S. A. 2011, 108, 10980. 21690347 10.1073/pnas.1106634108 PMC3131364

[20] S. Zou , B. Wang , C. Wang , Q. Wang , L. Zhang , Nanomedicine 2020, 15, 625. 32098564 10.2217/nnm-2019-0388

[21] Q. Xia , Y. Zhang , Z. Li , X. Hou , N. Feng , Acta Pharm. Sin. B 2019, 9, 675. 31384529 10.1016/j.apsb.2019.01.011 PMC6663920

[22] M. Gao , C. Liang , X. Song , Q. Chen , Q. Jin , C. Wang , Z. Liu , Adv. Mater. 2017, 29, 1701429. 10.1002/adma.201701429 28722140

[23] G. C. Terstappen , A. H. Meyer , R. D. Bell , W. Zhang , Nat. Rev. Drug Discovery 2021, 20, 362. 33649582 10.1038/s41573-021-00139-y

[24] W. Jia , H. Tian , J. Jiang , L. Zhou , L. Li , M. Luo , N. Ding , E. C. Nice , C. Huang , H. Zhang , Small 2023, 19, 2205354. 10.1002/smll.202205354 36399643

[25] R. D. Bell , M. D. Ehlers , Neuron 2014, 81, 1. 24411725 10.1016/j.neuron.2013.12.023

[26] S. L. van den Broek , V. Shalgunov , M. M. Herth , Biomater. Adv. 2022, 141, 213125. 36182833 10.1016/j.bioadv.2022.213125

[27] A. Zakeri , M. A. J. Kouhbanani , N. Beheshtkhoo , V. Beigi , S. M. Mousavi , S. A. R. Hashemi , A. Karimi Zade , A. M. Amani , A. Savardashtaki , E. Mirzaei , S. Jahandideh , A. Movahedpour , Nano Rev. Exp. 2018, 9, 1488497. 30410712 10.1080/20022727.2018.1488497 PMC6171788

[28] J. Wang , F. Meng , B. K. Kim , X. Ke , Y. Yeo , Biomaterials 2019, 217, 119296. 31254934 10.1016/j.biomaterials.2019.119296 PMC6670295

[29] Q. Jiang , Y. Liu , R. Guo , X. Yao , S. Sung , Z. Pang , W. Yang , Biomaterials 2019, 192, 292. 30465973 10.1016/j.biomaterials.2018.11.021

[30] A. N. Barclay , T. K. Van den Berg , Annu. Rev. Immunol. 2014, 32, 50. 10.1146/annurev-immunol-032713-120142 24215318

[31] A. V. Kroll , R. H. Fang , L. Zhang , Bioconjugate Chem. 2017, 28, 23. 10.1021/acs.bioconjchem.6b00569 PMC5471317 27798829

[32] R. Harati , A. S. Villégier , W. A. Banks , A. Mabondzo , J. Neuroinflammation 2012, 9, 273. 23253775 10.1186/1742-2094-9-273 PMC3547749

[33] M. C. Dalakas , H. Alexopoulos , P. J. Spaeth , Nat. Rev. Neurol. 2020, 16, 601. 33005040 10.1038/s41582-020-0400-0 PMC7528717

[34] R. Ejzemberg , M. H. Da Silva , L. Pinto , W. B. Mors , An. Acad. Bras. Cienc. 1999, 71, 273. 10412494

[35] K. S. Park , Biosens. Bioelectron. 2018, 102, 179. 29136589 10.1016/j.bios.2017.11.028 PMC7125563

[36] D. J. Patel , A. K. Suri , F. Jiang , L. Jiang , P. Fan , R. A. Kumar , S. Nonin , J. Mol. Biol. 1997, 272, 645. 9368648 10.1006/jmbi.1997.1281

[37] S. A. Liddelow , K. A. Guttenplan , L. E. Clarke , F. C. Bennett , C. J. Bohlen , L. Schirmer , M. L. Bennett , A. E. Münch , W. S. Chung , T. C. Peterson , D. K. Wilton , A. Frouin , B. A. Napier , N. Panicker , M. Kumar , M. S. Buckwalter , D. H. Rowitch , V. L. Dawson , T. M. Dawson , B. Stevens , B. A. Barres , Nature 2017, 541, 481. 28099414 10.1038/nature21029 PMC5404890

[38] S. A. Liddelow , B. A. Barres , Immunity 2017, 46, 957. 28636962 10.1016/j.immuni.2017.06.006

[39] V. Fleischer , J. Sieber , S. J. Fleischer , A. Shock , G. Heine , C. Daridon , T. Dörner , Arthritis Res. Ther. 2015, 17, 185. 26183319 10.1186/s13075-015-0686-2 PMC4504352

[40] H. Lian , L. Yang , A. Cole , L. Sun , A. C. Chiang , S. W. Fowler , D. J. Shim , J. Rodriguez‐Rivera , G. Taglialatela , J. L. Jankowsky , H. C. Lu , H. Zheng , Neuron 2015, 85, 101. 25533482 10.1016/j.neuron.2014.11.018 PMC4289109

[41] U. Kishore , S. K. Gupta , M. V. Perdikoulis , M. S. Kojouharova , B. C. Urban , K. B. Reid , J. Immunol. 2003, 171, 812. 12847249 10.4049/jimmunol.171.2.812

[42] L. B. Yang , R. Li , S. Meri , J. Rogers , Y. Shen , J. Neurosci. 2000, 20, 7505. 11027207 10.1523/JNEUROSCI.20-20-07505.2000 PMC6772855

[43] W. C. Pierre , P. L. P. Smith , I. Londono , S. Chemtob , C. Mallard , G. A. Lodygensky , Brain, Behav., Immun. 2017, 59, 333. 27596692 10.1016/j.bbi.2016.08.018

[44] S. A. Frautschy , F. Yang , M. Irrizarry , B. Hyman , T. C. Saido , K. Hsiao , G. M. Cole , Am. J. Pathol. 1998, 152, 307. 9422548 PMC1858113

[45] G. M. Cole , S. A. Frautschy , Trends Mol. Med. 2006, 12, 395. 16880006 10.1016/j.molmed.2006.07.002

[46] A. Chandra , G. Dervenoulas , M. Politis , J. Neurol. 2019, 266, 1293. 30120563 10.1007/s00415-018-9016-3 PMC6517561

[47] A. T. Du , N. Schuff , J. H. Kramer , S. Ganzer , X. P. Zhu , W. J. Jagust , B. L. Miller , B. R. Reed , D. Mungas , K. Yaffe , H. C. Chui , M. W. Weiner , Neurology 2004, 62, 422. 14872024 10.1212/01.wnl.0000106462.72282.90 PMC1820859

[48] B. T. Luk , C. M. Hu , R. H. Fang , D. Dehaini , C. Carpenter , W. Gao , L. Zhang , Nanoscale 2014, 6, 2730. 24463706 10.1039/c3nr06371b PMC3954976

[49] C. F. Rodrigues , N. Fernandes , D. de Melo‐Diogo , P. Ferreira , I.‐D. J. Correia , A. F. Moreira , Nanomedicine 2021, 16, 2569. 34854343 10.2217/nnm-2021-0270

[50] F. Bernard‐Patrzynski , M. A. Lécuyer , I. Puscas , I. Boukhatem , M. Charabati , L. Bourbonnière , C. Ramassamy , G. Leclair , A. Prat , V. G. Roullin , PLoS One 2019, 14, e0226302. 31851695 10.1371/journal.pone.0226302 PMC6919623

[51] N. L. Stone , T. J. England , S. E. O'Sullivan , Front. Cell. Neurosci. 2019, 13, 230. 31244605 10.3389/fncel.2019.00230 PMC6563620

[52] Y. Luo , Q. Wen , S. Yang , Y. Feng , T. Tan , Biomed. Chromatogr. 2020, 34, e4762. 31760665 10.1002/bmc.4762

