# Supplementary material for: Study on the Role of an Erythrocyte Membrane‐Coated Nanotheranostic System in Targeted Immune Regulation of Alzheimer's Disease
Source: Adv Sci (Weinh). 2023 Apr 19;10(18):2301361. doi: 10.1002/advs.202301361 (PMC10288270; doi:10.1002/advs.202301361)
Supplement: Supplementary file 1 — Supporting Information [file ADVS-10-2301361-s001.pdf]

Supporting information：

Study on the role of an erythrocyte membrane-coated nanotheranostic system in targeted immune regulation of Alzheimer's disease

Yanrong Su, Yufen Huang, Qinjie Kou, Lu Lu, Haiye Jiang, Xisheng Li, Rong Gui, Rong Huang, Xueyuan Huang, Jinqi Ma, Jian Li, Xinmin Nie**

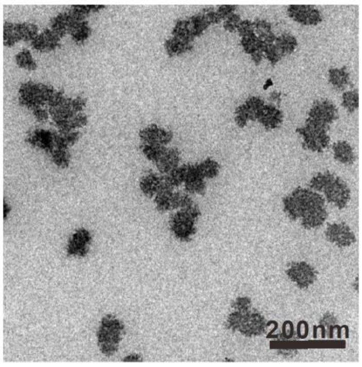

Figure S1: TEM image of ZC-PEI, scale bar: 200 nm.

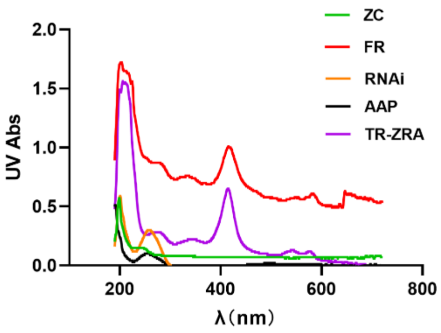

Figure S2: UV–vis absorbance of NPs.

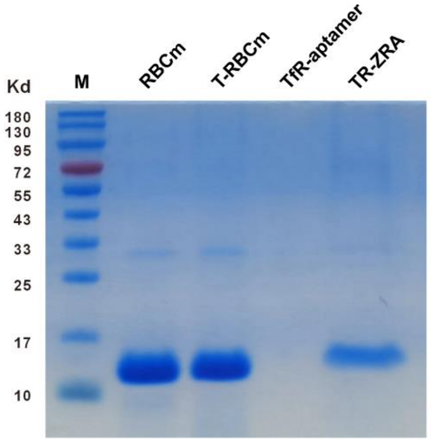

Figure S3: SDS-PAGE protein analysis of RBCm, T-RBCm, TfR-aptamer, TR-ZRA.

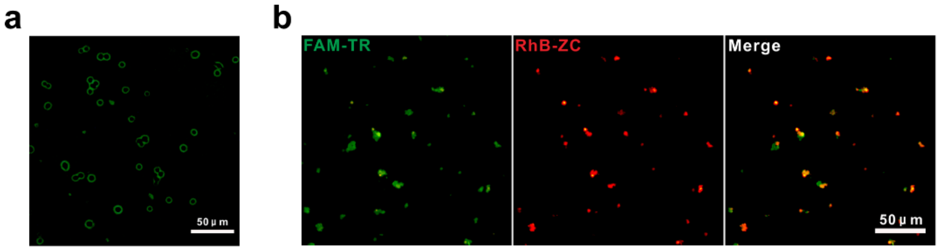

Figure S4: a) Fluorescence image of TR, showing that obvious green fluorescence on the surround of RBCm. b) The colocalization fluorescence image of FAM-TR and RhB-ZC. Scale bar :50 μm.

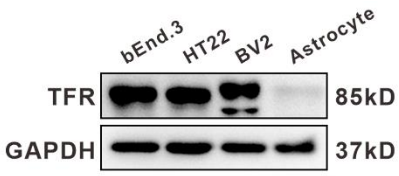

Figure S5: Western blot assay evaluated transferrin receptor expression on bEnd.3, HT22, BV2 and Astrocyte.

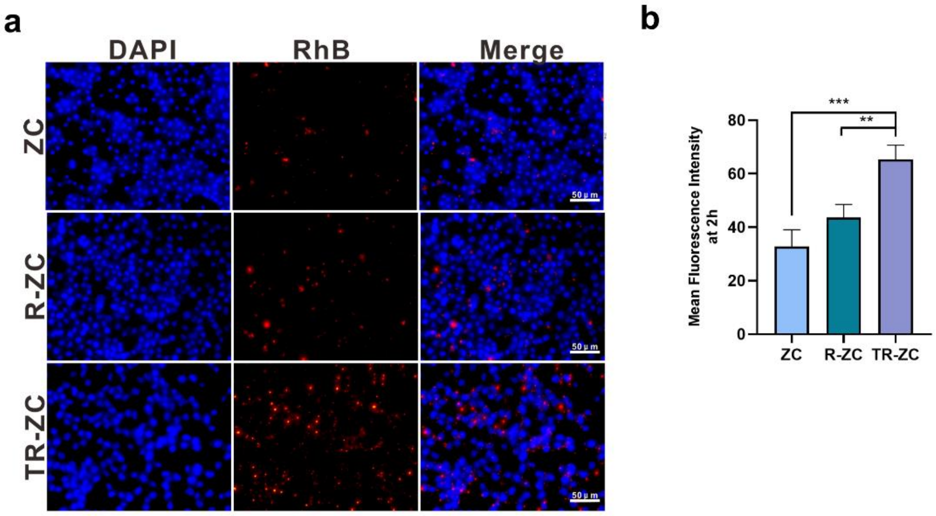

Figure S6: a) The cellular uptake of NPs in bEnd.3 cells after 2 h incubation and b) quantification of fluorescence intensity from (a), scale bar :50 μm. $**p<0.01$, $***p<0.001$ determined by one-way ANOVA and Tukey post hoc tests. Data are presented as mean ± SD (n=3).

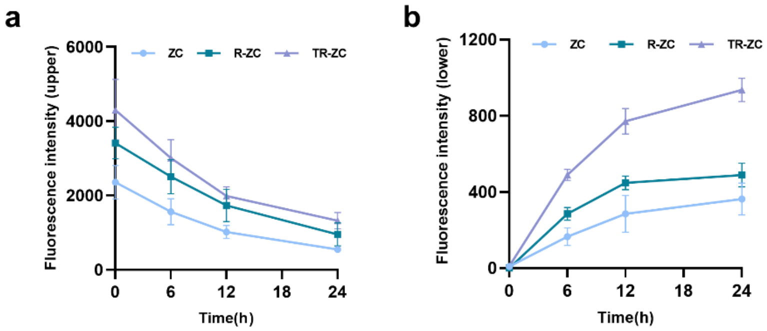

Figure S7: The time-dependent change of fluorescence intensity in the a) upper medium and b) lower medium in vitro BBB model. Data are presented as mean ± SD (n=3).

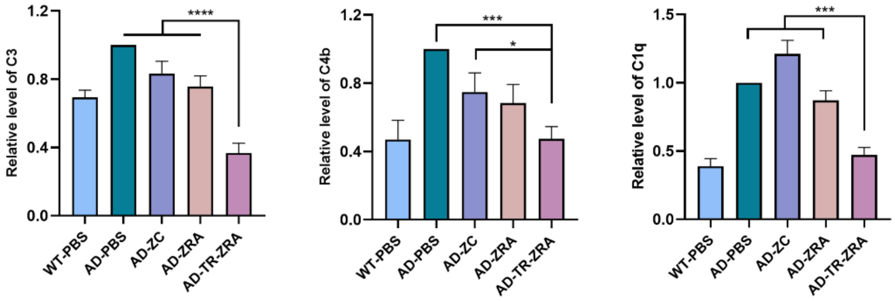

Figure S8: The quantified results of complement component C3, C4b, C1q from western blotting in the brain tissue of WT and mice. Aβ-PBS group was as control. *p<0.05, ***p<0.001, ****p<0.0001 determined by one-way ANOVA and Tukey post hoc tests. Data are presented as mean ± SD (n=3).

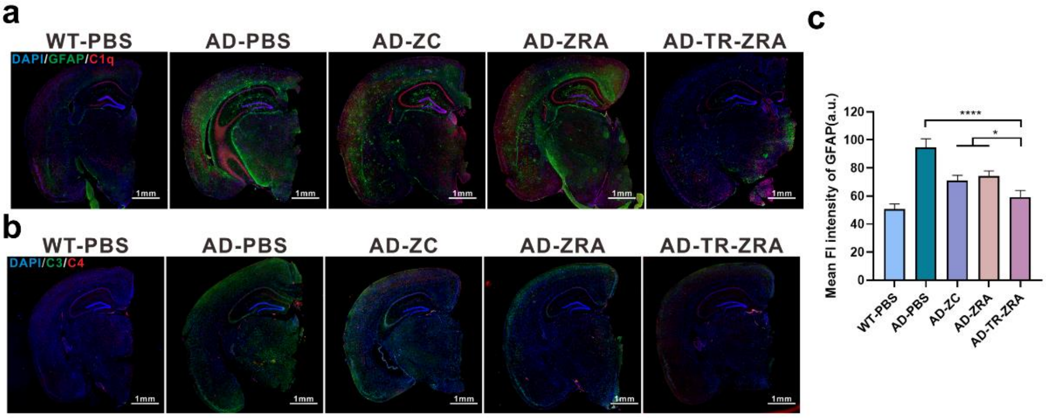

Figure S9: a) The immune fluorescence staining of GFAP and C1q, b) C3 and C4b in the whole brain environment, scale bar:1mm. c) Quantified mean fluorescence intensity of GFAP from (a). *p<0.05, ****p<0.0001 determined by one-way ANOVA and Tukey post hoc tests. Data are represented as the mean ± SD (n=3).

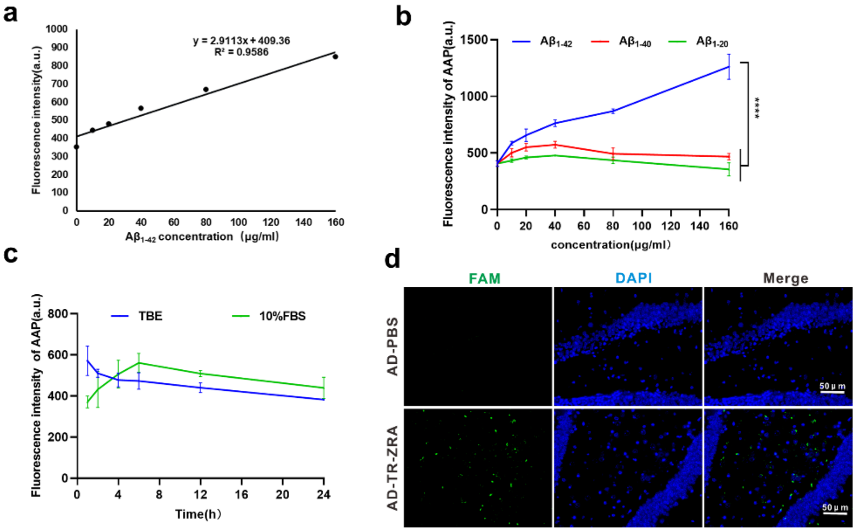

Figure S10: Aβ detection by Aβ aptamer. a) Standard curve on $A\beta_{1-42}$-AAP at 37 °C. b) Fluorescence intensity change of AAP after incubation with Aβ aggregates ($A\beta_{1-42}$, $A\beta_{1-40}$, $A\beta_{1-20}$) of different concentrations at 37 °C for 2 h. c) Fluorescence intensity change of AAP incubating with $A\beta_{1-42}$ in TBE and 10% FBS at 37 °C within 24 h. d) Fluorescence images in mice brain sections in the AD-PBS and AD-TR-ZRA groups (Aβ aptamer: green, DAPI: blue); scale bar:50 µm. **** $p < 0.0001$ determined by one-way ANOVA and Tukey post hoc tests. Data are represented as the mean ± SD (n=3).

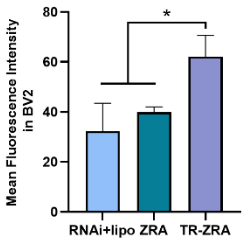

Figure S11. Quantification of mean fluorescence intensity in BV2 after incubation with mCherry labeled RNAi+lipo, ZRA or TR-ZRA at 37 °C for 24 h. *$p<0.05$ determined by one-way ANOVA and Tukey post hoc tests. Data are represented as the mean ± SD (n=3).

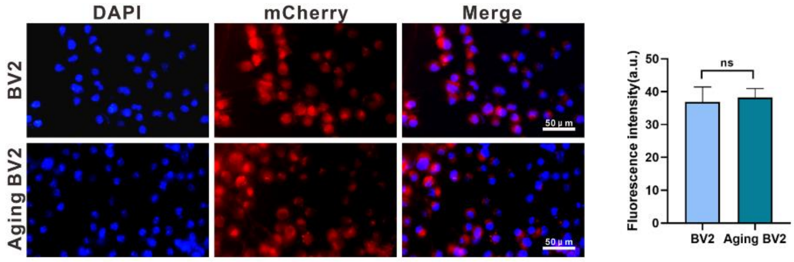

Figure S12. Representative fluorescence images and quantification of mean fluorescence intensity in BV2 and aging BV2 after incubation with TR-ZRA at 37 °C for 24 h. Statistical analysis was performed using t-test and ns means no significance. Data are represented as the mean ± SD (n=3).

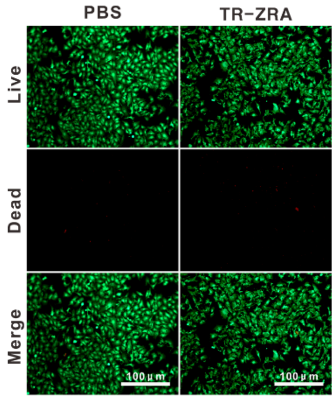

Figure S13:The Live/Dead staining of HT22 after incubation with TR-ZRA for 24 h at 37 °C; scale bar 100 µm.

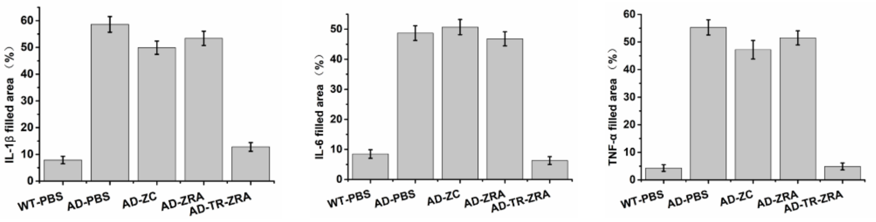

Figure S14. Quantification of inflammatory cytokines (IL-1β, TNF-α, IL-6) expressed in WT and AD mice brain tissue from Figure 8c. Data are represented as the mean ± SD (n=3).

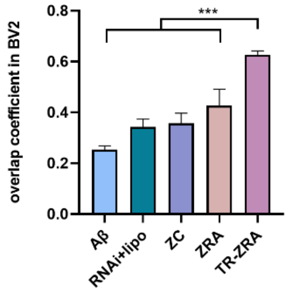

Figure S15: The overlap coefficient lyso-tracker and Aβ in BV2. *** $p < 0.001$ determined by one-way ANOVA and Tukey post hoc tests. Data are represented as the mean ± SD (n=3).

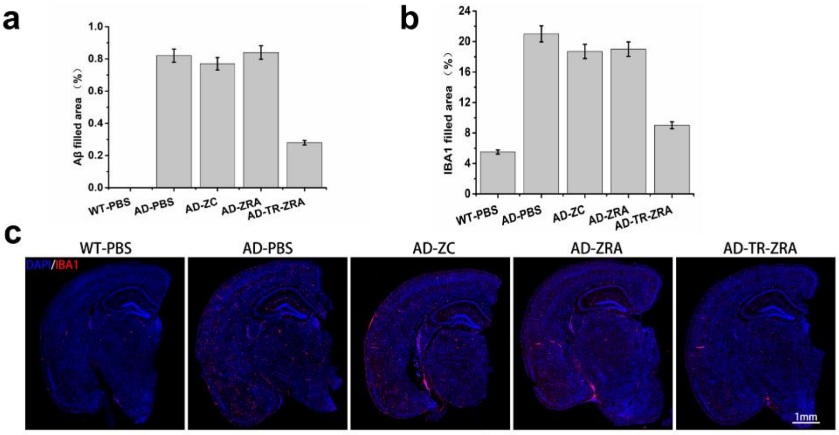

Figure S16: a) The quantification of Aβ plaque in the brain tissue of each group of mice. b) The quantification of IBA1 and c) fluorescence image in the whole brain tissue of each group of mice. Scale bar: 1mm. Data are represented as the mean ± SD (n=3).

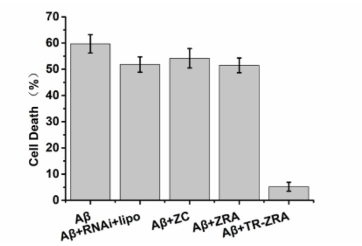

Figure S17: Quantification of the Live/Dead staining of HT22 after incubation with BV2 medium for 24 h at 37 °C. Data are represented as the mean ± SD (n=3).

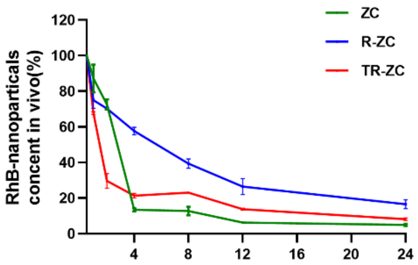

Figure S18: Pharmacokinetic changes within 24 h in mice after injection of nanoparticles. Data are represented as the mean ± SD (n=3).

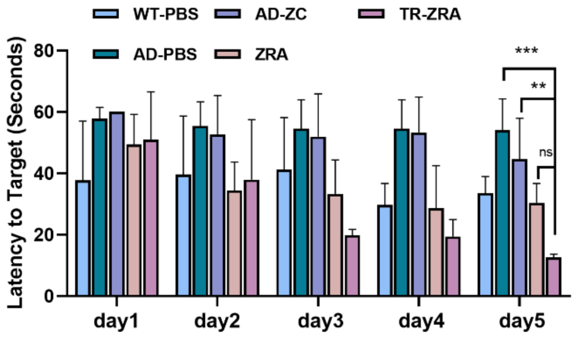

Figure S19: Memory capability improved in vivo. Changes in latency during the first 5 days of training in MWM. ** $p < 0.01$, *** $p < 0.001$ determined by one-way ANOVA and Tukey post hoc tests. Data are presented as mean ± SD (n=3).

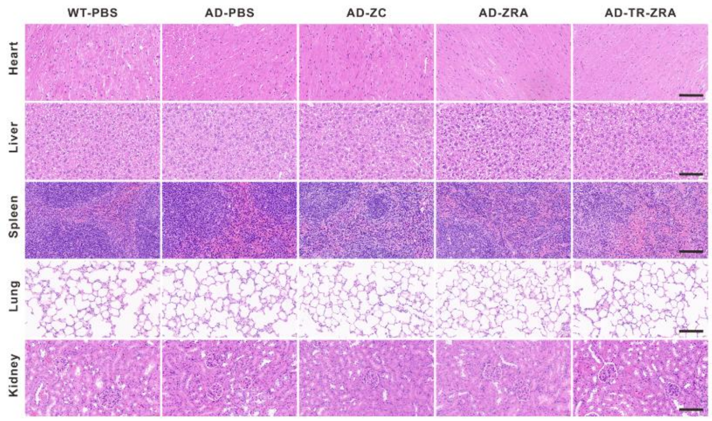

Figure S20: Representative HE staining images of various organs in WT and AD mouse; scale bar :100μm.
